# A tissue-based draft map of the murine MHC class I immunopeptidome

**DOI:** 10.1038/sdata.2018.157

**Published:** 2018-08-07

**Authors:** Heiko Schuster, Wenguang Shao, Tobias Weiss, Patrick G.A. Pedrioli, Patrick Roth, Michael Weller, David S. Campbell, Eric W. Deutsch, Robert L. Moritz, Oliver Planz, Hans-Georg Rammensee, Ruedi Aebersold, Etienne Caron

**Affiliations:** 1 Department of Immunology, Interfaculty Institute for Cell Biology, University of Tübingen, Tübingen 72076, Germany; 2 Immatics Biotechnologies GmbH, 72076 Tübingen, Germany; 3 German Cancer Research Center (DKFZ) partner site Tübingen, 72076 Tübingen, Germany; 4 Department of Biology, Institute of Molecular Systems Biology, ETH Zurich, Zurich 8093, Switzerland; 5 Department of Neurology and Brain Tumor Center, University Hospital Zurich and University of Zurich, 8091 Zurich, Switzerland; 6 Institute for Systems Biology, Seattle, Washington 98109, USA; 7 Faculty of Science, University of Zurich, 8006 Zurich, Switzerland

**Keywords:** Immunology, Systems biology

## Abstract

The large array of peptides presented to CD8+ T cells by major histocompatibility complex (MHC) class I molecules is referred to as the MHC class I immunopeptidome. Although the MHC class I immunopeptidome is ubiquitous in mammals and represents a critical component of the immune system, very little is known, in any species, about its composition across most tissues and organs *in vivo*. We applied mass spectrometry (MS) technologies to draft the first tissue-based atlas of the murine MHC class I immunopeptidome in health. Peptides were extracted from 19 normal tissues from C57BL/6 mice and prepared for MS injections, resulting in a total number of 28,448 high-confidence H2D^b^/K^b^-associated peptides identified and annotated in the atlas. This atlas provides initial qualitative data to explore the tissue-specificity of the immunopeptidome and serves as a guide to identify potential tumor-associated antigens from various cancer models. Our data were shared via PRIDE (PXD008733), SysteMHC Atlas (SYSMHC00018) and SWATH Atlas. We anticipate that this unique dataset will be expanded in the future and will find wide applications in basic and translational immunology.

## Background & Summary

T cells are an essential cell type for next-generation vaccines and immunotherapies^1^. T cells recognize antigens in the form of short peptides presented by MHC (human leucocyte antigen [HLA] in humans) molecules — collectively referred to as the MHC ligandome/peptidome or immunopeptidome^2–4^. Robust and comprehensive immunopeptidomic profiling of primary cells and tissues is therefore of great importance for the development of effective T-cell based immunotherapies^5^.

The cellular immunopeptidome is composed of thousands of MHC-associated peptides—each peptide ranging in abundance between approximately 1 and 10,000 copies per cell^6^. The immunopeptidome can be divided into two main categories: the MHC class I and the MHC class II immunopeptidome. The latter is composed of peptides of 10-25 amino acids in length that are mainly presented on a subset of professional antigen presenting cells. In contrast, the class I immunopeptidome is composed of peptides presented on the surface of virtually any nucleated cell. Class I peptides are generally of 8-12 amino acids in length^7,8^. In mammals, the composition of the immunopeptidome is complicated by the high diversity of allelic forms^9^. Each allelic form can present a different set of peptides that are characterized by the presence of allele-specific anchor residues, known as MHC binding motif^10^. In humans, more than 17,600 alleles have been documented (IPD-IMGT/HLA Database; December 2017; http://hla.alleles.org/alleles/index.html) and up to six class I and eight class II alleles can be expressed per cell in each individual. In mouse, >200 alleles are expressed among the most commonly used mouse strains (http://www.imgt.org/IMGTrepertoireMHC/Polymorphism/haplotypes/mouse/MHC/Mu_haplotypes.html), and up to two class I and two class II alleles can be expressed per cell in each mouse strain. Even though the composition of the immunopeptidome is highly complex in nature, the deployment of robust technology platforms has facilitated the deciphering of the immunopeptidome at increasing depth and robustness^5,11^. MS is most widely used due to its capability of identifying and quantifying MHC-associated peptides in an accurate, systematic and unbiased manner^12^. In fact, many immunopeptidomics studies have demonstrated the ability of MS workflows to identify thousands of MHC-associated peptides from various biological sources in human, mouse and other species^13–23^. Those studies led to a better and systematic understanding of antigen presentation and provided direct physical evidence for the existence of tumor-specific peptides. Nevertheless, only a handful of studies have reported detailed information about the composition of the immunopeptidome in healthy cells and tissues. More specifically, immunopeptidomic analyses of normal thymic cells^24,25^, peripheral blood mononuclear cells^26^, and spleen and lymph nodes^3,27^ have been documented. Thus, basic information about the identity, abundance and distribution of MHC-associated peptides across normal tissues and organs in healthy humans, mice or other species is still largely missing in the literature.

**Figure 1:**
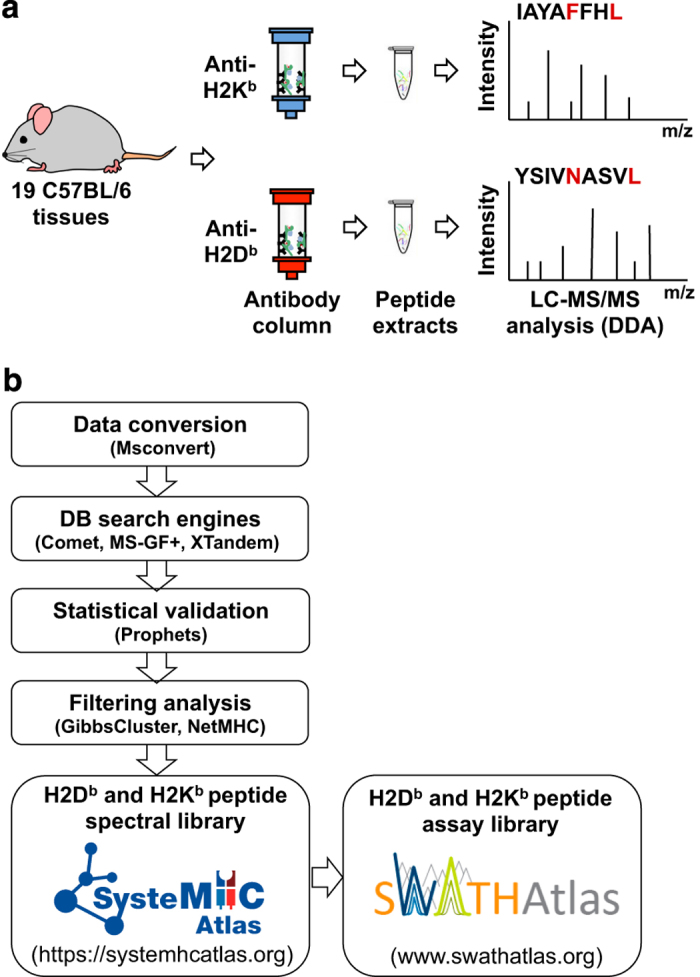
Schematic overview of the experimental and computational workflow used to generate and analyze the data. (**a**) 19 different tissues from C57BL/6 mice were extracted (Table 1) (**Annotation Table,** Data Citation 1). H2D^b^ and H2K^b^-associated peptides were isolated independently by immunoaffinity purification using the monoclonal antibodies B22-249.R1 and Y-3, respectively. Eluted peptides were identified by different LC-MS/MS systems in DDA mode. (**b**) MS output files were converted, searched, and statistically validated using the indicated software tools. The identified peptides were then clustered (GibbsCluster v.1) and annotated by length and predicted MHC binding affinity (NetMHC v.4). The final list of high-confidence MHC-associated peptides were used to build high-quality H2D^b^- and H2K^b^-specific peptide spectral and assay libraries, which were deposited and shared via SysteMHC Atlas and SWATH Atlas, respectively.

Open and comprehensive reference maps in life sciences, including tissue-based maps, are increasingly beneficial for the scientific community^28–32^. Similarly, the creation of comprehensive maps of the immunopeptidome in human, mouse, and other species would be of great value for both understanding health and diagnosing, monitoring and treating immune diseases^5^. Given the advances in MS technology over the last decade, the availability of protocols for the isolation MHC-associated peptides from multiple species and tissue types, and the relatively less complex composition of the immunopeptidome in mouse models (in comparison with humans), we reasoned that the time was ripe to initiate a systematic effort to draft the first MS-based atlas of the murine MHC class I immunopeptidome in health using a commonly used mouse strain. To this end, we used data-dependent acquisition (DDA) MS to generate immunopeptidomic data from 19 tissues of healthy C57BL/6 mice. They express both H2D^b^ and H2K^b^ class I molecules (Fig. 1a). We also mapped the immunopeptidome of four C57BL/6-derived cancer cell lines and used an open and evolving computational pipeline to process the data. Several stringent filters to generate a list of high-confidence H2D^b^/K^b^ class I peptides for individual tissues and cell lines were applied. All raw/unfiltered MS data as well as H2D^b^/K^b^ peptide spectral libraries — which consist of consensus spectra calculated from repeat measurement of the same peptide sequence — are made publicly available for re-use and re-processing by the community for in-depth interrogation of the dataset (Fig. 1b). In summary, the present study provides a unique resource for basic and translational immunologists to navigate the baseline immunopeptidome in mouse. An open reference map of the murine immunopeptidome in health is valuable for i) basic and translational immunologists to rapidly identify disease-specific MHC peptide antigens — through comparison of peptides found in the reference map versus those identified in disease cells — and ii) computational scientists to access a rich source of data to support technical benchmarking of future studies to develop or test new algorithms for immunopeptidomic analyses. In addition, this reference map, together with its connection with SWATH Atlas, lays down the foundation to perform robust quantitative analysis of the murine immunopeptidome using next-generation SWATH/Data-independent acquisition (DIA)-MS technologies^26,33^.

## Methods

### Mouse tissues and cell lines

Adrenal gland, bladder, bone marrow, brain, colon, heart, kidney, liver, lung, ovary, pancreas, small intestine, skin, spinal cord, spleen, stomach, testis, thymus, and uterus were extracted from C57BL/6 male or female mice (**Annotation Table,** Data Citation 1). The EL4, LLC1 (LL/2) and B16F10 cell lines were obtained from ATCC. The GL261 cell line was obtained from DSMZ. All cell lines were cultured in DMEM with GlutaMAX-1 supplemented with 100 U/mL penicillin, 100 μg/mL streptomycin and 10% fetal bovine serum. B16F10 was treated with IFNγ (200 U/mL) for 24 h to increase the cell surface expression of H2D^b^ and H2K^b^ molecules.

### Isolation of MHC class I-associated peptides

H2D^b^- and H2K^b^-associated peptides were isolated by a conventional immunoaffinity purification method using the monoclonal antibodies B22-249.R1 and Y-3, respectively^22^. For generating the tissue-based map of the murine MHC class I immunopeptidome, the tissue/organs from five to six mice were pooled together before isolating MHC-peptide complexes for any given tissue (**Annotation Table,** Data Citation 1). For each cell line used in this study, ~10^9^ cells were grown before isolating MHC-peptide complexes. The cell surface abundance of MHC proteins was also quantified for each cell line using the QIFIKIT quantification flow cytometric assay, as previously described^34^.

### DDA mass spectrometry

Fragment ion spectra of the respective MHC class I peptide preparations were acquired on an Orbitrap Fusion Lumos and/or a Triple TOF 5600+ (see below) operated in DDA mode. For retention time (RT) normalization and spectral library generation, peptides from the iRT Kit (Biognosys AG, Schlieren, Switzerland) were added to the samples prior to MS injection according to vendor instructions^35^ (Data Citation 2).

For Lumos data (**Annotation Table,** Data Citation 1), peptides were separated on an Acclaim PepMap RSLC C18 column (250 mm x 75 um i.d., 2 Å particle size; ThermoFisher Scientific) using a flow rate of 300 nl min-1 and a linear gradient of 4–29.6% aqueous ACN (with 0.1% formic acid) in 120 min. Full mass spectra were acquired with the Orbitrap analyser operated at a resolving power of 120,000 (at m/z 200). MS/MS spectra were acquired in both HCD and CID mode with a normalized collision energy of 27%. Precursors were selected in the "top speed" mode with a cycle time of 3 s. Fragment ions (charge state 2-6+) were accumulated up to an AGC target value of 50,000 with a maximum injection time of 54 ms and the option "Inject ions for all available parallelizable time" enabled, and were detected in the Orbitrap analyzer at a resolution of 30,000 (at m/z 200). Dynamic exclusion was enabled for 30 s after a selection event with a tolerance of±10 p.p.m.

For Triple TOF 5600+ data, (**Annotation Table,** Data Citation 1). Samples were separated on an Eksigent nanoLC system coupled with an AB SCIEX Triple TOF 5600^+^ System. The samples were separated in a 75 μm-diameter PicoTip emitter (New Objective, Woburn, MA) packed with 20 cm of Magic 3 μm, 200 Å C18 AQ material (Bischoff Chromatography, Leonberg, Germany). The loaded peptides were eluted from the column at a flow rate of 300 nl/min and a linear gradient of 2–35% aqueous ACN (0.1% formic acid) over 120 min. The mass spectrometer was operated in DDA top20 mode, with 500 and 150 ms acquisition time for the MS1 and MS2 scans respectively, and 20 s dynamic exclusion. MS/MS spectra were acquired in CID mode. Rolling collision energy with a collision energy spread of 15 eV was used for fragmentation.

### Database search engines, statistical validation, high-confidence filters and spectral library generation

Raw mass spectrometry files were converted into the mzXML format by msConvert^36^. The mzXML files were then individually searched using Comet^37^, MSGF^38^ and X!Tandem^39^ against the full non-redundant, canonical mouse genome as annotated by the UniProtKB/Swiss-Prot (2014_02) with 20,270 ORFs and appended iRT peptides and reversed decoy sequences. Oxidation at methionine residues was the only variable modification allowed. We used default search settings for all the engines with the following key parameters: Precursor tolerance was set to ±20 p.p.m., high accuracy fragment ion tolerance was set to ±0.02 Da for Comet and 20 p.p.m. for X!Tandem, and digestion specificity was set to unconstrained. The search identifications from different search engines were then combined and statistically scored using PeptideProphet and iProphet within the TPP (4.8.0), as previously described^40^. The probabilities estimated by iProphet was cut at 1% FDR. Then, all 8, 9-mers (for H2K^b^) and all 9-11-mers (for H2D^b^) were clustered using GibbsCluster (v1.0)^41^ to visualize MHC binding motifs enriched in the dataset. To select the final list of high-confidence H2D^b^- and H2K^b^-associated peptides, strict cut-off criteria were applied: FDR 1% (peptide-spectrum match level); 8–9 and 9–11 amino acids in length for H2K^b^ and H2D^b^ peptides, respectively; and IC_50_<500 nM (NetMHC v4.0). Spectral libraries were generated by SpectraST using the list of high-confidence H2D^b^/K^b^ peptides, with default consensus library building parameters, as previously described^26^. H2D^b^- and H2K^b^-specific peptide spectral libraries were then combined and generated on the peptide atlas level that contains consensus spectra of peptides from different samples. For a given allele-specific spectral library, the same peptide ions generated under various fragmentation methods (CID Orbitrap, CID TOF and HCD) were specified and kept separated as different library entries. The generated spectral libraries were further converted into TraML format and archived in SWATH Atlas for SWATH/DIA-MS analysis.

## Data Records

The accession number for the DDA-MS data (raw and centroided mzXML and identified peptides in pepXML report) used to generate the spectral libraries have been deposited to the ProteomeXchange Consortium (http://proteomecentral.proteomexchange.org) via the PRIDE partner repository^42^ with the dataset identifier PXD008733 (Data Citation 2). Raw and mzXML files are also accessible via the SysteMHC Atlas repository (https://systemhcatlas.org/)^40^ with the dataset identifier SYSMHC00018. The H2D^b^- and H2K^b^-associated peptide spectral libraries (SpectraST format) and assay libraries (CSV, TraML) are available for different SWATH/DIA-MS data analysis tools at SWATH Atlas (http://www.swathatlas.org/). Spectral libraries are also accessible at SysteMHC Atlas (https://systemhcatlas.org/speclibs). The lists of all peptides (unfiltered and filtered) are available in figshare (https://figshare.com/s/5436fdcdb908000a49d5) (Data Citation 1).

## Technical Validation

### MS-based identification of high-confidence H2D^b^- and H2K^b^-associated peptides in 19 tissues of healthy mice

The draft map of the murine MHC class I immunopeptidome was generated from 19 different C57BL/6 tissues extracted under steady-state conditions (Fig. 1,Table 1 and Methods). H2D^b^- and H2K^b^-peptide complexes were isolated by immunoaffinity purification using the B22-249.R1 and the Y-3 antibody, respectively. Peptides were acid-eluted and acquired in DDA mode using different MS instruments (Table 1). Following acquisition of data from 280 MS runs, ∼4.8 million MS/MS spectra were searched using a uniform and well-tested computational pipeline^26,40^ (Fig. 1b) and yielded assignments of 681,357 and 850,396 peptide ions with iProphet probability *P*≥0.9 and *P*>0.0, respectively.

Next, we considered all 7–14 mers identified at FDR 1%, resulting in a total number of 81,058 peptides (Supplementary Figure 1) (List of unfiltered H2D^b^ peptides (7–14 mers), Data Citation 1) (List of unfiltered H2K^b^ peptides (7–14 mers), Data Citation 1). We then applied very strict confidence filters (see Methods) to remove potential non-MHC binding contaminant peptides. As an example, we observed that after filtering, 72% of all 9-mer H2D^b^ peptides and 81% of all 8-mer H2K^b^ peptides (FDR 1%) identified from spleen tissue were predicted to have a strong MHC binding affinity with IC_50_<500 nM (Supplementary Figure 2a). Similarly, 80% of all 9-mer H2D^b^ peptides and 80% of all 8-mer H2K^b^ peptides (FDR 1%) identified from heart tissue were predicted to have a strong MHC binding affinity with IC_50_<500 nM (Supplementary Figure 2m). These data and similar data from other tissue types indicate that the antibodies that were used in this study are relatively specific and the proportion of high-confidence H2D^b^/K^b^-associated peptides that were identified from different tissue types was generally high and varied only slightly. Data to calculate the proportion of high-confidence H2D^b^/K^b^-associated peptides per tissue and cell type are available in (List of unfiltered H2D^b^ peptides (7-14 mers), Data Citation 1) (List of unfiltered H2K^b^ peptides (7-14 mers), Data Citation 1). Longer peptides (i.e.>11 amino acids for H2D^b^ and>9 amino acids for H2K^b^) or peptides predicted to bind H2D^b^/K^b^ with a lower affinity (IC_50_>500 nM) were considered in this study as low-confidence H2D^b^/K^b^ peptides–although they might still be genuine H2D^b^/K^b^-associated peptides–and were therefore not included for downstream analysis and spectral library generation.

**Figure 2:**
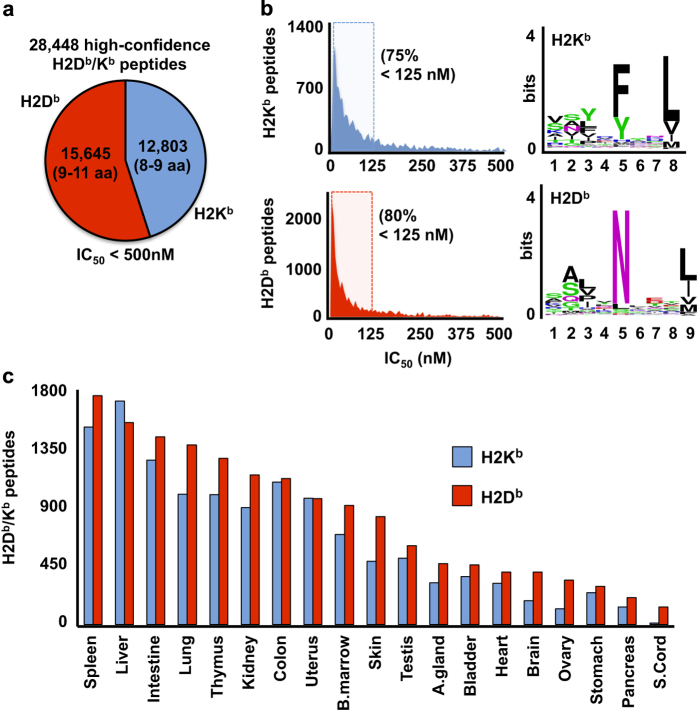
Identifications of high-confidence H2D^b^/K^b^-associated peptides from 19 normal mouse tissues. (**a**) Pie chart indicating the total number of high-confidence H2D^b^- and H2K^b^-associated peptides that were identified across all tissues. (**b**) Graphs showing the high proportion of high-confidence H2K^b^- (upper panel) and H2D^b^- (lower panel) peptides with a predicted MHC binding affinity (IC_50_) below 125 nM. The binding motifs for H2K^b^- and H2D^b^-associated peptides were illustrated. (**c**) Histogram showing the distribution of high-confidence H2D^b^/K^b^-associated peptides identified per mouse tissue.

After filtering the whole dataset, the number of high-confidence H2D^b^/K^b^-associated peptides identified per tissue demonstrated a high variability that ranged from 146 (spinal cord tissue) to 3,263 (spleen tissue) with an average number of 1,497 peptides (Fig. 2c). The different amounts of tissues (in grams) used for immunoprecipitation as well as sample handling may have contributed to this large difference in the number of identified peptides. Nevertheless, we observed that the number of high-confidence peptides identified per tissue generally correlated with the abundance of MHC class I proteins previously reported from the same mouse tissues (Supplementary Figure 3)^43^. Overall 15,645 (2,693 unique) high-confidence H2D^b^-associated peptides (FDR 1%, 9–11 amino acids, IC_50_<500 nM) and 12,803 (2,594 unique) H2K^b^-associated peptides (FDR 1%, 8–9 amino acids, IC_50_<500 nM) were identified (Fig. 2). The identified peptides mapped to 4,050 of the mouse UniProtKB/Swiss-Prot proteins. Of note, 36.4 and 27.4% of all the high-confidence H2D^b^- and H2K^b^-associated peptides were not shared across tissues but were rather exclusively detected in one particular tissue (Table 2). In contrast, a relatively small proportion of the measured H2D^b^ immunopeptidome (0.2%) and H2K^b^ immunopeptidome (1.9%) was shared across all the 19 tissues (Table 2). For instance, the H2K^b^-associated peptides INFDFPKL and VNFEFPEF were found in all the 19 tissues whereas the H2D^b^-associated peptides AAITNGLAM and HSVINQAVM were found exclusively in the brain. It is important to emphasize, however, that the proportion of tissue-shared and tissue-specific peptides mentioned above were not calculated from quantitative and normalized values. In fact, it is very likely that the low coverage overlap described above would have increased significantly if larger amounts of tissues–for those expressing lower levels of MHC molecules–would have been used for immunoprecipitation. In future studies, it will be important to consider the absolute abundance of MHC molecules per tissue type and adjust/normalize the amounts of starting material accordingly to investigate in a more rigorous manner the tissue-specificity of the MHC class I immunopeptidome. Additional factors such as sample handling, yield of the immunoaffinity purification procedure per tissue type, and limits of detection (LOD) and quantification (LOQ) of mass spectrometers used for identifying MHC-associated peptides would also need to be considered. Taken together, these results delineate the first draft map of the murine H2D^b^/K^b^ class I immunopeptidome in health and provide initial qualitative data to further explore the tissue-specificity of the immunopeptidome.

### A reference map of the murine MHC class I immunopeptidome in health guides identification of potential tumor-associated antigens (TAAs)

**Figure 3:**
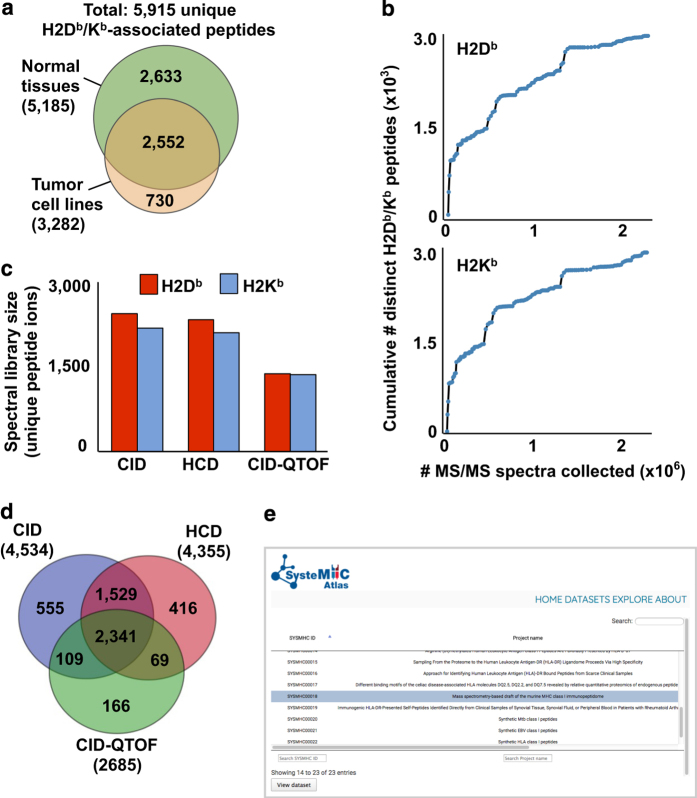
Analysis of high-quality H2D^b^/K^b^-specific peptide spectral libraries generated from healthy mouse tissues and tumor cell lines. (**a**) Venn diagram showing the overlap between high-confidence H2D^b^/K^b^-associated peptides identified from 19 healthy tissues and 4 tumor cell lines used in this study. (**b**) Cumulative number of MS/MS spectra acquired versus cumulative number of distinct high-confidence H2D^b^/K^b^ peptides identified. Each data point represents an added injection/MS experiment, and the experiments are presented in a chronological order of data acquisition (see Order of injection for H2Kb peptides; Data Citation 1 and Order of injection for H2Db peptides; Data Citation 1). (**c**) Histogram indicating the number of distinct peptide ions that were generated from normal tissues using different fragmentation methods (i.e. Orbitrap CID, HCD and CID-QTOF). High-quality MHC allele- and fragmentation-specific peptide spectral libraries were generated using SpectraST. (**d**) Venn diagram showing the overlap between high-confidence H2D^b^/K^b^-associated peptides generated from normal tissues using different fragmentation methods. (**e**) Screenshot of SysteMHC Atlas (https://systemhcatlas.org/). The raw MS output files, the peptide sequences and the spectral libraries are accessible at SysteMHC Atlas with the dataset identifier SYSMHC00018.

The rapid and robust identification of TAAs or tumor-specific antigens is relevant for the development of cancer vaccines, and the generation of a reference map of the MHC class I immunopeptidome in health supports identification of such peptide antigens^5,34,44^. In this regard, we compared the list of peptides found in the 19 healthy mouse tissues to those found in several *in vitro* tumor models. More specifically, we profiled the H2D^b^/K^b^ immunopeptidome of four different cancer types from four widely used C57BL/6 tumor-derived cell lines: 1) EL4 cells (lymphoma), 2) LLC1 cells (Lewis lung carcinoma), 3) GL261 cells (malignant glioma) and 4) B16F10 cells (melanoma). In summary, 3,282 unique high-confidence H2D^b^/K^b^-associated peptides were identified in the four tumor cell lines, 2,552 peptides were shared between the healthy tissues and the tumor cell lines, and 730 (22%) peptides were exclusively observed in the tumor cell lines (Fig. 3a) (**List of high-confidence H2D**^**b**^ **peptides,** Data Citation 1**) (List of high-confidence H2K**^**b**^ **peptides,** Data Citation 1). The presence of tumor cell line-specific peptides was also noted. For instance, 28 peptides and 49 peptides were exclusively identified in GL261 and B16F10 cells, respectively (Supplementary Figure 4). Those peptides might be classified as glioma- and melanoma-associated antigens, respectively, if further tested and validated. Thus, a reference map of the murine immunopeptidome in health guides identification of potential TAAs in model cell lines. We envision that a comprehensive reference map of the murine immunopeptidome in health will find application in tumor immunology and beyond, e.g. in immunopathology to identify a wide variety of disease-specific peptide antigens.

### H2D^b^/K^b^ peptide spectral libraries saturation analysis

Comprehensive and robust quantitative analysis of the immunopeptidome is important to 1) identify new immunotherapeutic targets, 2) better understand the relationship between T cells and MHC-presenting cells, and 3) potentially identify immunopeptidomic biomarker signatures in normal and disease cells from sample cohorts. Building high-quality peptide spectral libraries was demonstrated to be an efficient procedure to support robust quantitative analysis of immunopeptidomes using advanced MS techniques, i.e. SWATH/DIA^26,33,40^. To estimate the status of our initial mapping effort and to support robust quantitative analysis of the murine MHC class I immunopeptidome, we created H2D^b^/K^b^-specific peptide spectral libraries and we plotted the cumulative number of distinct H2D^b^/K^b^ peptides as a function of the number of MS2 spectra acquired on the mass spectrometer (Fig. 3b). Each data point on the curve represents an added injection/experiment, and the experiments are presented in chronological order of data acquisition (**Order of injection for H2K**^**b**^ **peptides,** Data Citation 1**) (Order of injection for H2D**^**b**^ **peptides,** Data Citation 1). The graphs indicate that new H2D^b^/K^b^ peptides were continuously identified as additional MS/MS spectra were collected, suggesting that new peptides will probably be discovered in future experiments, as saturation has not been reached using the presently available technology. Therefore, collecting more data from new experiments (e.g. additional cell lines, additional primary tissues, new experimental conditions, new protocols and MS technologies) will be needed to enable comprehensive and robust quantitative analysis of the murine MHC class I immunopeptidome in the future. In addition, we anticipate that absolute quantitative analysis of immunopeptidomes—i.e. absolute quantification of MHC molecules as well as absolute and systematic quantification of individual MHC-associated peptides per cell and tissue type—will become essential to rigorously assess the completeness of this initial mapping effort.

### Sharing H2D^b^/K^b^ peptidomic data via SysteMHC Atlas

We anticipate that the dataset generated in this study will be widely used by basic and translational immunologists as well as computational mass spectrometrists. Therefore, an important goal here is to share our immunopeptidomics MS-related data at many different levels of processing. Specifically, we provide raw and converted mzXML files, lists of high-confidence peptides (iProphet results) and H2D^b^/K^b^ peptide spectral libraries, all available for download from the SysteMHC Atlas (Fig. 3**) (H2D**^**b**^**/K**^**b**^ **peptides used for spectral library generation,** Data Citation 1).

The SysteMHC Atlas is a new public data repository that serves as a community resource toward the generation of high-quality comprehensive maps of immunopeptidomes and the support of consistent measurements of immunopeptidomic sample cohorts^40^. Until now, the SysteMHC Atlas contains 540 sample/context- and 39 MHC allele-specific peptide spectral libraries (37 HLA and 2 H2^b^), all available for download from the web interface. Moreover, the H2D^b^- and H2K^b^-specific peptide spectral libraries generated in this study were both converted into TraML files for robust quantitative analysis of immunopeptidomes using SWATH/DIA-MS, as described previously^26,45^. TraML files are available at SWATH Atlas (www.swathatlas.org). Notably, three separate fragmentation-specific libraries were created: 1) CID and 2) HCD using the Orbitrap Fusion Lumos, and 3) CID-QTOF using the Triple TOF 5600+ (Fig. 3c). Different fragmentation methods are complementary and can be used to enhance the identification success rate of MHC-associated peptides and to thus increase immunopeptidome coverage (Fig. 3d). More importantly, the CID-QTOF-, CID- and HCD-specific spectral libraries support the high-throughput targeted analysis of SWATH/DIA immunopeptidomic data generated by these different fragmentation methods.

In the future, we foresee that continuous development of SysteMHC Atlas for effective sharing and re-analysis of immunopeptidomic datasets will be key to comprehensively define the composition and complexity of the murine immunopeptidome. For instance, we envisage that re-analysis of raw MS data using advanced peptide sequencing algorithms might unveil the presence of non-canonical MHC-associated peptides, e.g. proteasome-spliced peptides^46,47^ (https://www.biorxiv.org/content/biorxiv/early/2018/03/26/288209.full.pdf), which would be of particular relevance for the development of peptide-based vaccines and immunotherapies in precision medicine^48^.

## Usage notes

The lists of peptides provided in figshare (Data Citation 1) may differ from the ones available at SysteMHC Atlas (https://systemhcatlas.org)^40^. The SysteMHC computational pipeline used to generate the peptide lists is subjected to periodic upgrades and the resulting data may be different from the original publication. To ensure reproducibility of the results, we have introduced a database versioning system and the current build version is 180409 (year/month/date). Current and past builds can also be downloaded at: https://systemhcatlas.org/Builds_for_download/. This information is also available in the ‘ABOUT’ section of the SysteMHC Atlas website.

## Additional information

**How to cite this article**: Schuster, H. *et al*. A tissue-based draft map of the murine MHC class I immunopeptidome. *Sci. Data* 5:180157 doi: 10.1038/sdata.2018.157 (2018).

**Publisher’s note**: Springer Nature remains neutral with regard to jurisdictional claims in published maps and institutional affiliations.

## Supplementary Material



Supplementary Information

## Figures and Tables

**Table 1:** Overview of normal tissues and tumor cell lines used to generate the draft map and spectral libraries.

**Sample type**	**Amount (g)**	**MS instrument**	**Nbr. MS injections**	
Spleen	Tissue	1.91	Lumos + TTOF	26
Liver	Tissue	15.98	Lumos + TTOF	22
Intestine	Tissue	12.42	Lumos + TTOF	27
Lung	Tissue	7.71	Lumos + TTOF	20
Thymus	Tissue	1.57	Lumos + TTOF	15
Kidney	Tissue	6.33	Lumos + TTOF	22
Colon	Tissue	5.53	Lumos + TTOF	22
Uterus	Tissue	1.06	Lumos + TTOF	17
Bone marrow	Tissue	NA	Lumos	4
Skin	Tissue	3.84	Lumos	4
Testis	Tissue	2.94	Lumos + TTOF	13
Adrenal gland	Tissue	0.64	Lumos	4
Bladder	Tissue	0.29	Lumos + TTOF	12
Heart	Tissue	0.91	Lumos + TTOF	18
Brain	Tissue	9.4	Lumos + TTOF	24
Ovary	Tissue	0.36	Lumos + TTOF	12
Stomach	Tissue	1.51	Lumos + TTOF	10
Pancreas	Tissue	0.13	Lumos	4
Spinal cord	Tissue	NA	Lumos + TTOF	4
Lymphoma	EL4 cell line	10^9^ cells	Lumos + TTOF	10
Lewis lung carcinoma	LLC1 cell line	10^9^ cells	Lumos	14
Malignant glioma	GL261 cell line	10^9^ cells	Lumos	10
Melanoma	B16F10 cell line	10^9^ cells	Lumos	20
Total				334
The Orbitrap Fusion Lumos (Lumos) and the Triple TOF 5600 (TTOF) were used to generate the data in both HCD/CID and CID mode, respectively. The full sample annotation is provided in (**Annotation Table,** Data Citation 1). All data files are available at (Data Citation 1) and (Data Citation 2). NA indicates that the amount of tissue was not available.				

**Table 2:** Number of tissue-specific (1 tissue) and tissue-shared (2-19 tissues) H2K^b^- and H2D^b^-associated peptides identified in this study.

**Tissue categories**	**H2K**^**b**^ **peptides (%)**	**H2D**^**b**^ **peptides (%)**
1 tissue	979 (36.4)	710 (27.4)
2 tissues	377 (14.0)	313 (12.1)
3 tissues	219 (8.1)	205 (7.9)
4 tissues	141 (5.2)	129 (5.0)
5 tissues	113 (4.2)	120 (4.6)
6 tissues	100 (3.7)	133 (5.1)
7 tissues	104 (3.9)	136 (5.2)
8 tissues	97 (3.6)	109 (4.2)
9 tissues	82 (3.0)	97 (3.7)
10 tissues	75 (2.8)	88 (3.4)
11 tissues	81 (3.0)	73 (2.8)
12 tissues	53 (2.0)	80 (3.1)
13 tissues	56 (2.1)	61 (2.4)
14 tissues	43 (1.6)	45 (1.7)
15 tissues	55 (2.0)	53 (2.0)
16 tissues	41 (1.5)	63 (2.4)
17 tissues	37 (1.4)	56 (2.2)
18 tissues	35 (1.3)	75 (2.9)
19 tissues	5 (0.2)	48 (1.9)
Total	2693 (100)	2594 (100)
The proportion of peptides per tissue categories is indicated in parenthesis.		
