# Supplementary material for: A tissue-based draft map of the murine MHC class I immunopeptidome
Source: Sci Data. 2018 Aug 7;5:180157. doi: 10.1038/sdata.2018.157 (PMC6080492; doi:10.1038/sdata.2018.157)
Supplement: Supplementary Information [file sdata2018157-s2.pdf]

Table of contents for Supplementary Figures 1 – 4.

Schuster et al. A tissue-based draft map of the murine MHC class I immunopeptidome.

Supplementary Figure 1. ………………………………………….……..p. 1

Supplementary Figure 2a – 2w. ………………..……………...p. 2 – 24

Supplementary Figure 3. ……..…………………………..…..……….p. 25

Supplementary Figure 4. ..……………………......…………….……p. 26

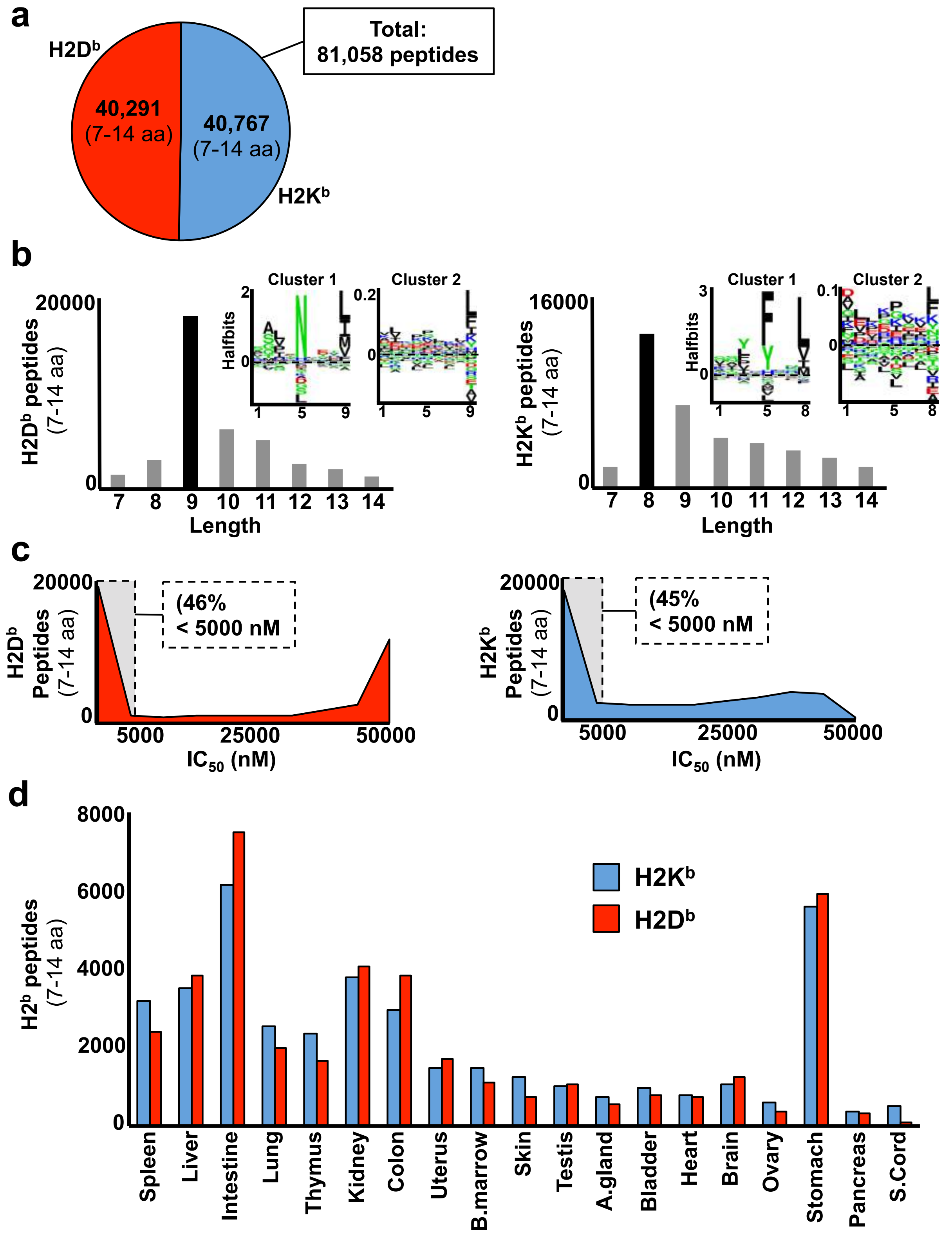

Supplementary Figure 1. Analysis of all 7-14 mers identified from 19 mouse tissues. (**a**) Pie chart showing the total number of peptides (7-14 mers) that were identified at FDR 1% before applying the filtering steps (note that the post-filtering pie chart is shown in Figure 2a). (**b**) Histograms showing the length distribution of all identified $H2D^b$ (left panel) and $H2K^b$ (right panel) peptides. GibbsCluster (v.1) analysis enabled generation of distinct peptide clusters; $H2D^b$ 9-mers and $H2K^b$ 8-mers are shown. (**c**) Graphs showing distribution of predicted MHC binding affinity values (IC_{50} obtained from NetMHC v.4) of the identified 7-14 mers, i.e. before applying the filtering steps. The proportion of peptides with an IC_{50} below 5000 nM is shown in parenthesis. (**d**) Histogram showing the total number of peptides (7-14 mers) identified per tissue before applying the filtering steps.

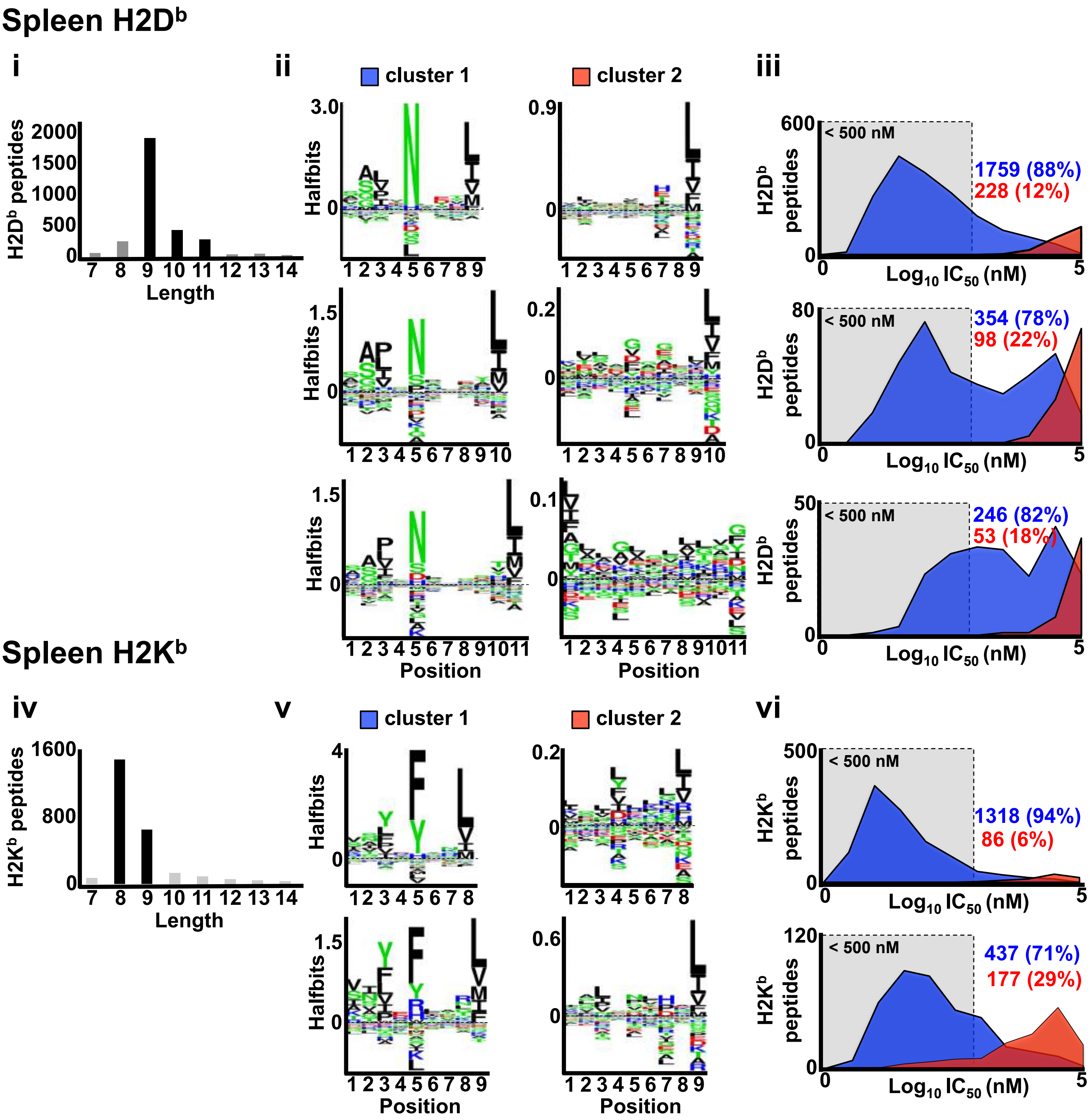

Supplementary Figure 2a. Analysis of all 7-14 mers identified at 1% FDR from spleen tissue. (**i** and **iv**) Histograms showing the length distribution of the identified H2D^b and H2K^b peptides. (**ii** and **v**) GibbsCluster analysis enabled generation of distinct peptide clusters; 9-, 10-, 11-mers (H2D^b), and 8-, 9-mers (H2K^b) are shown. (**iii** and **vi**) Graphs showing distribution of predicted MHC binding affinity values (IC_{50} obtained from NetMHC) of the identified peptides: (iii) H2D^b 9-mers (upper panel), H2D^b 10-mers (middle panel), H2D^b 11-mers (lower panel), (vi) H2K^b 8-mers (upper panel), and H2K^b 9-mers (lower panel). The number of peptides in cluster 1 (blue) and cluster 2 (red) is indicated and the respective proportion of peptides per cluster is in parenthesis. Peptides with an IC_{50} below 500 nM are in the grey box.

Liver H2D^b

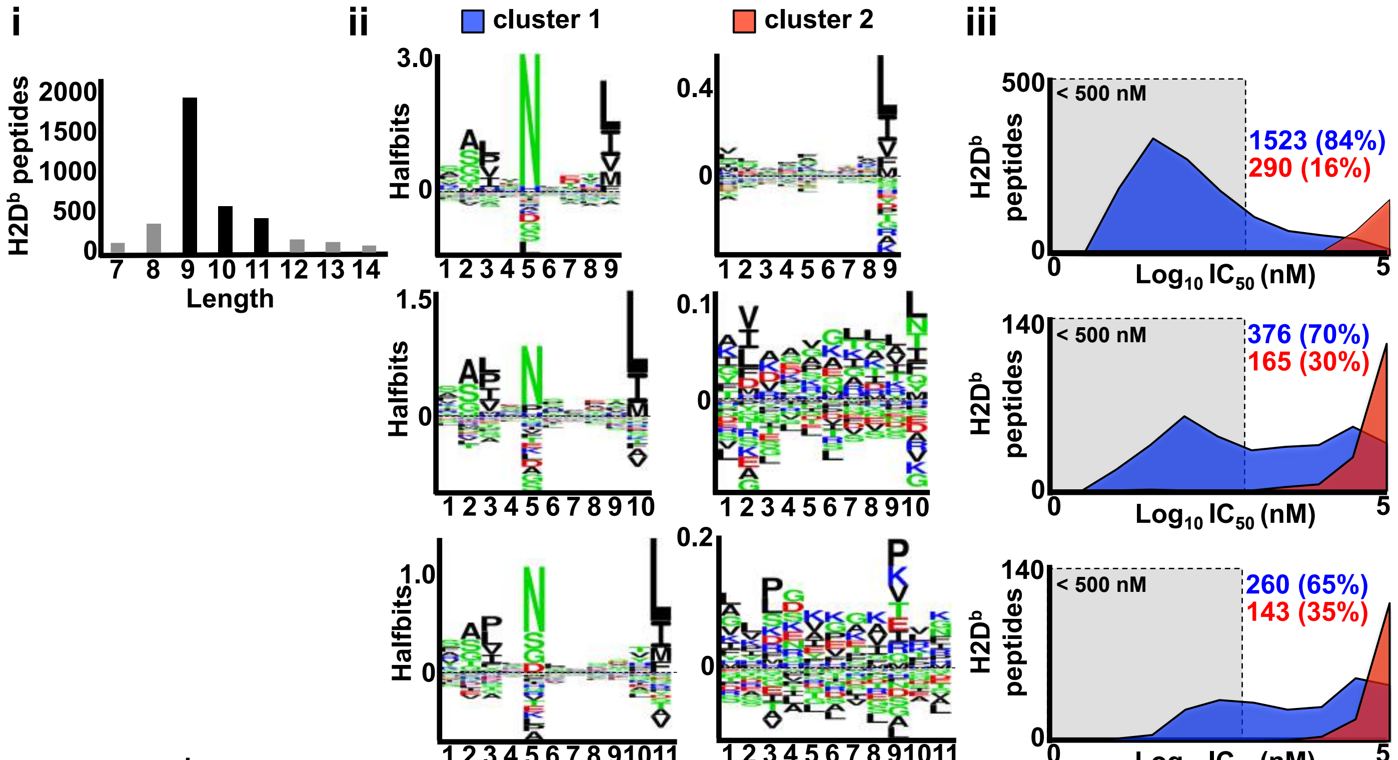

Liver H2K^b

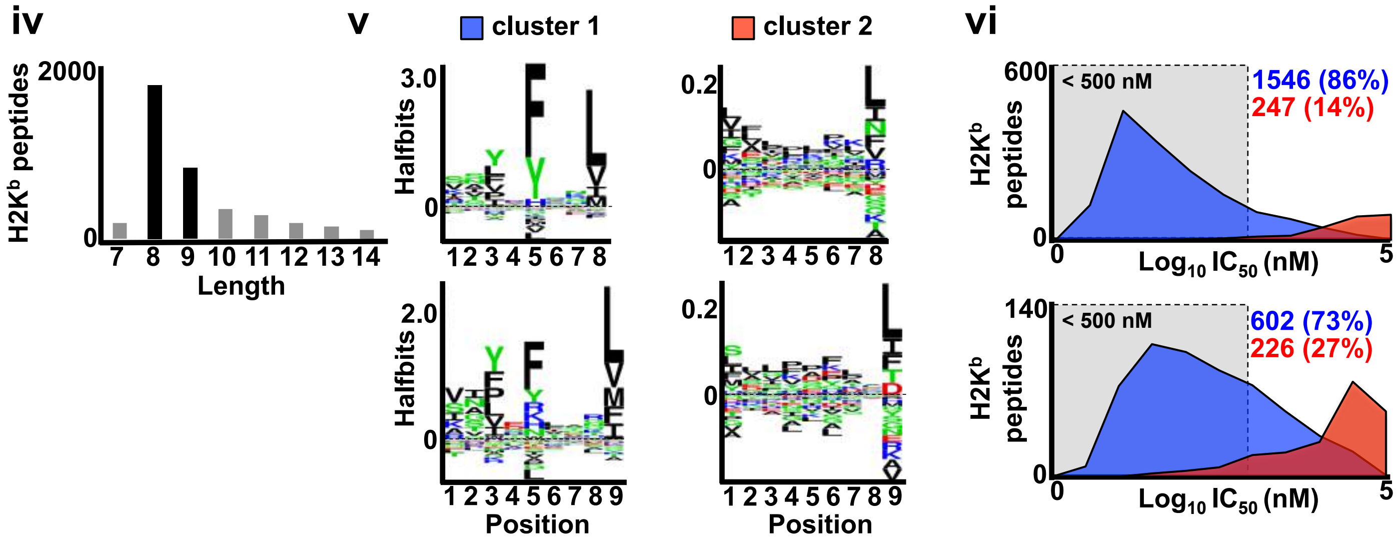

Supplementary Figure 2b. Analysis of all 7-14 mers identified at 1% FDR from liver tissue. See legend of Supplementary Figure 2a for detailed description of the panels' figure.

Small intestine H2D^b

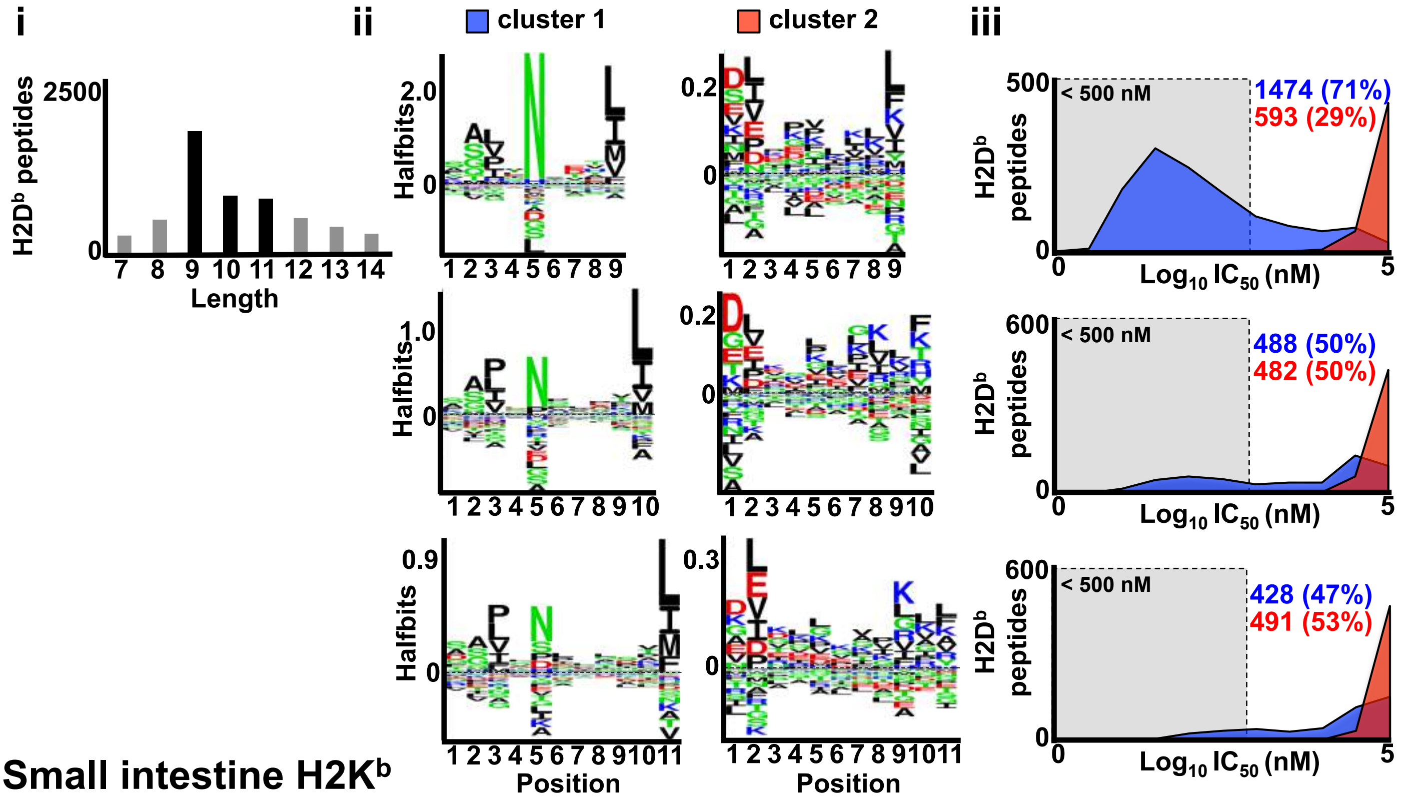

Small intestine H2K^b

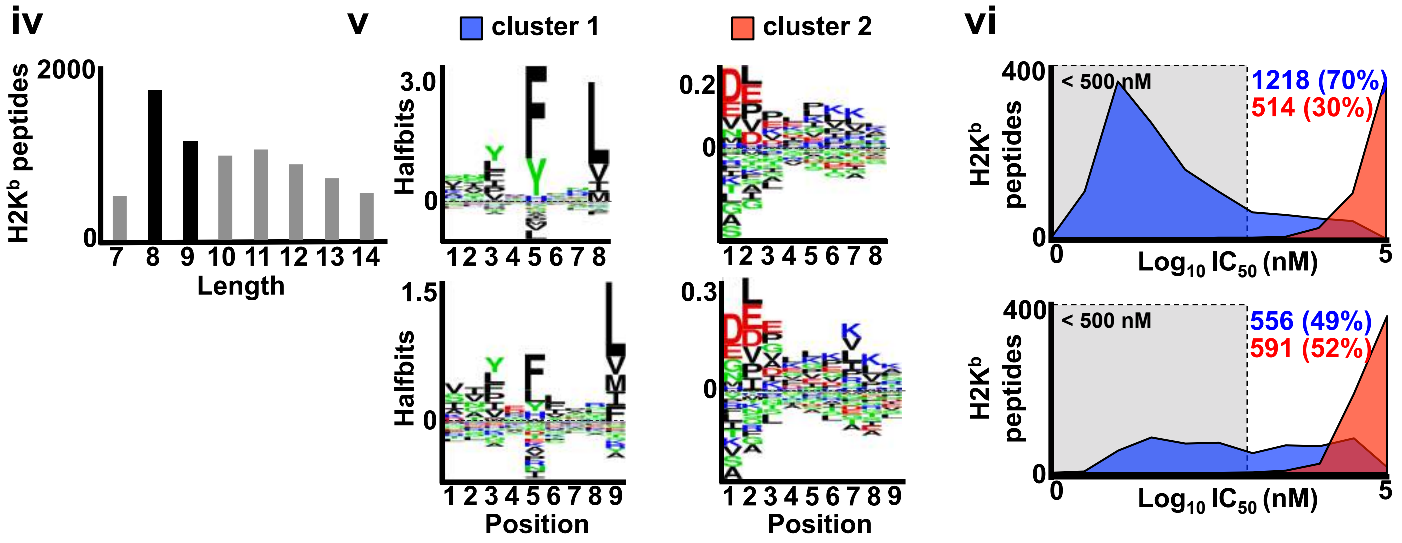

Supplementary Figure 2c. Analysis of all 7-14 mers identified at 1% FDR from small intestine tissue. See legend of Supplementary Figure 2a for detailed description of the panels' figure.

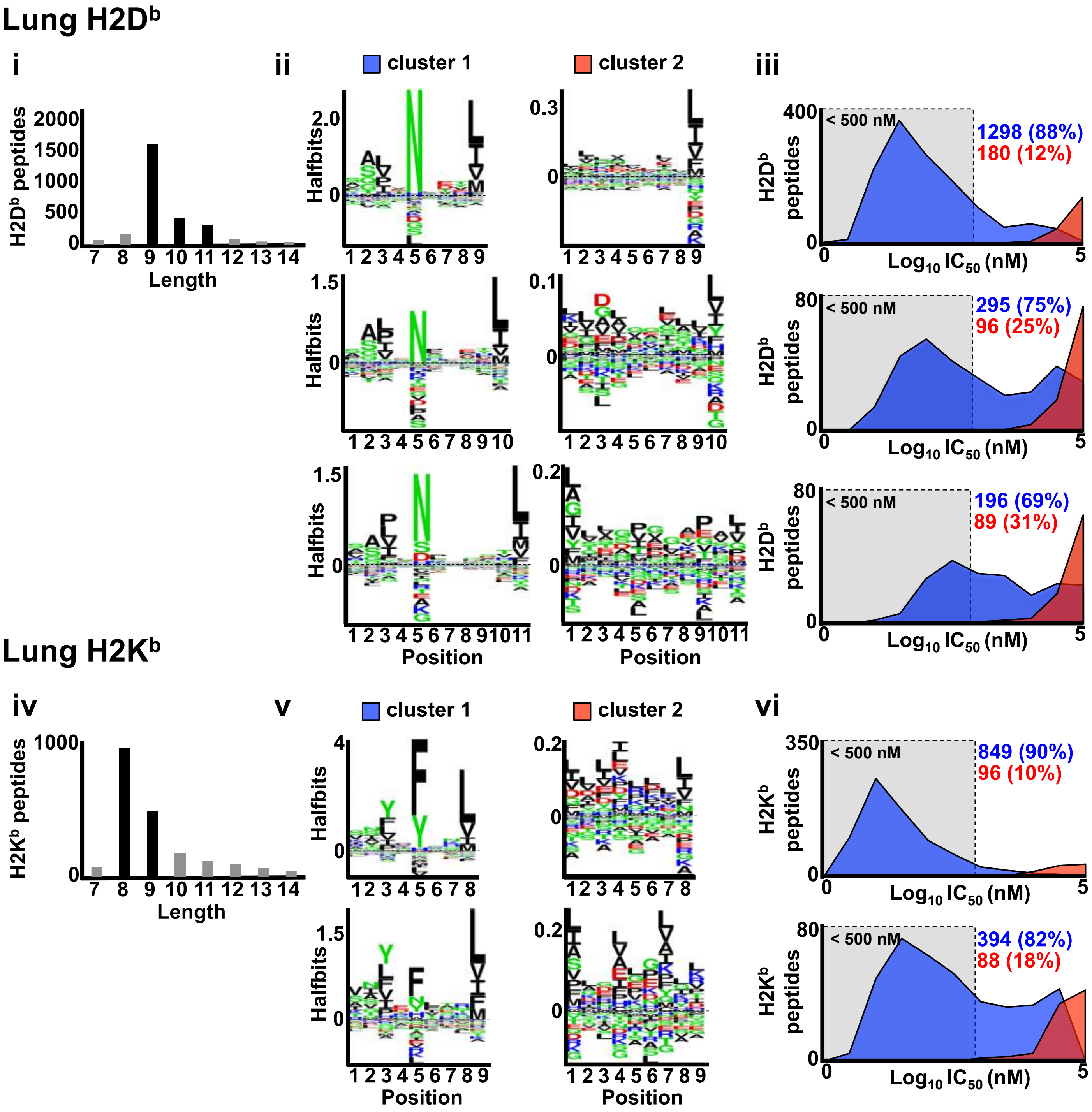

Supplementary Figure 2d. Analysis of all 7-14 mers identified at 1% FDR from lung tissue. See legend of Supplementary Figure 2a for detailed description of the panels' figure.

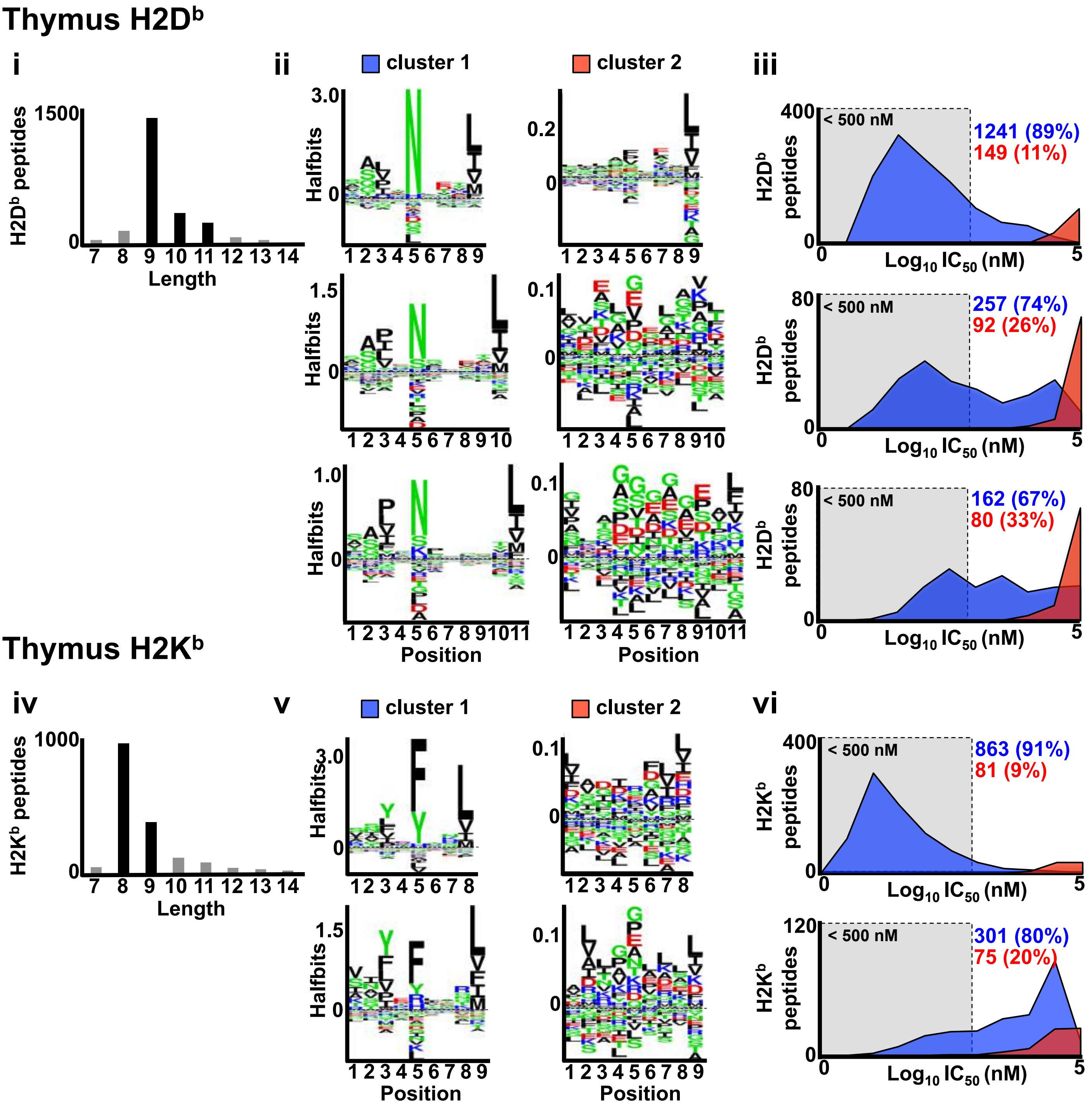

Supplementary Figure 2e. Analysis of all 7-14 mers identified at 1% FDR from thymus tissue. See legend of Supplementary Figure 2a for detailed description of the panels' figure.

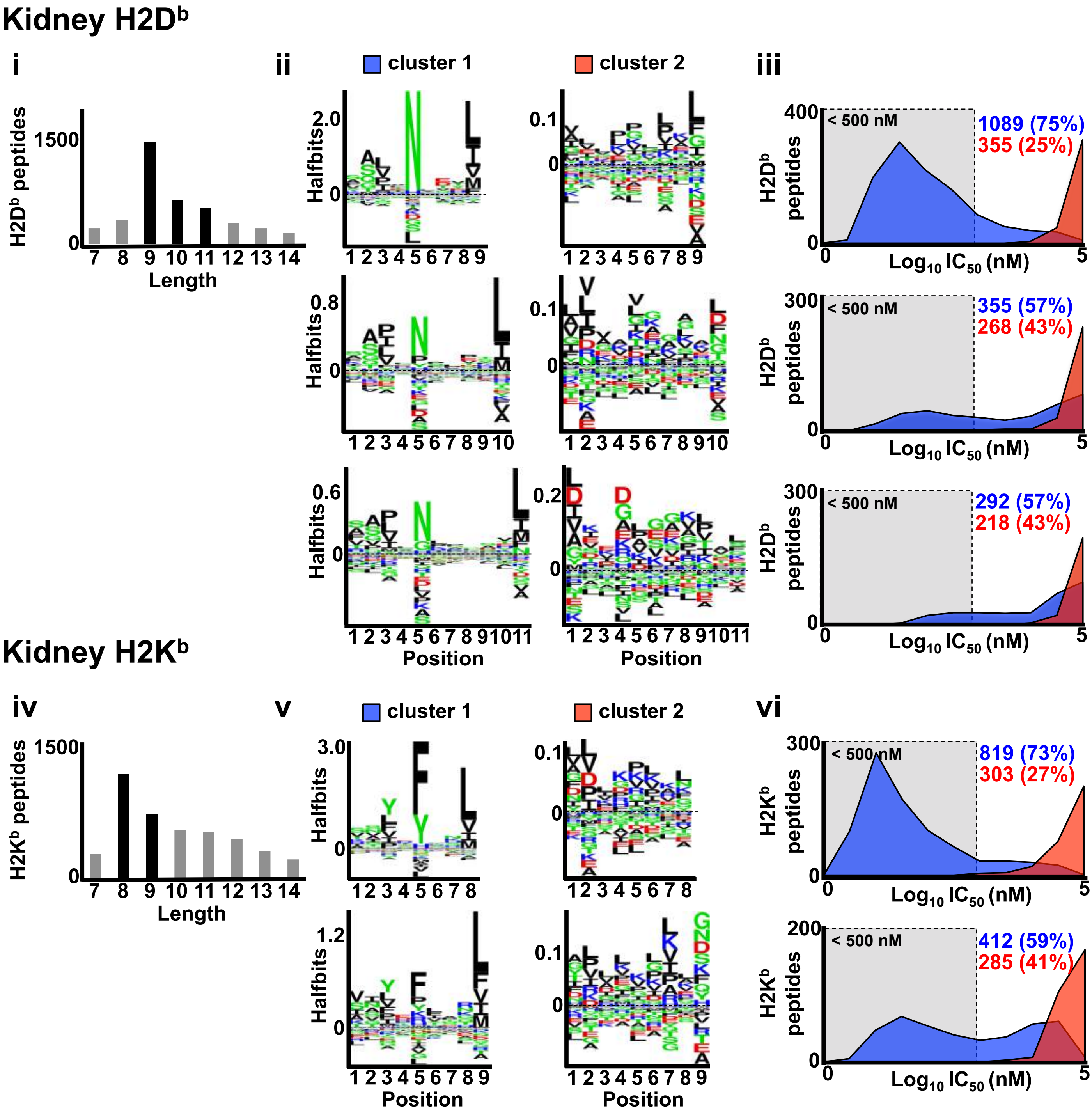

Supplementary Figure 2f. Analysis of all 7-14 mers identified at 1% FDR from kidney tissue. See legend of Supplementary Figure 2a for detailed description of the panels' figure.

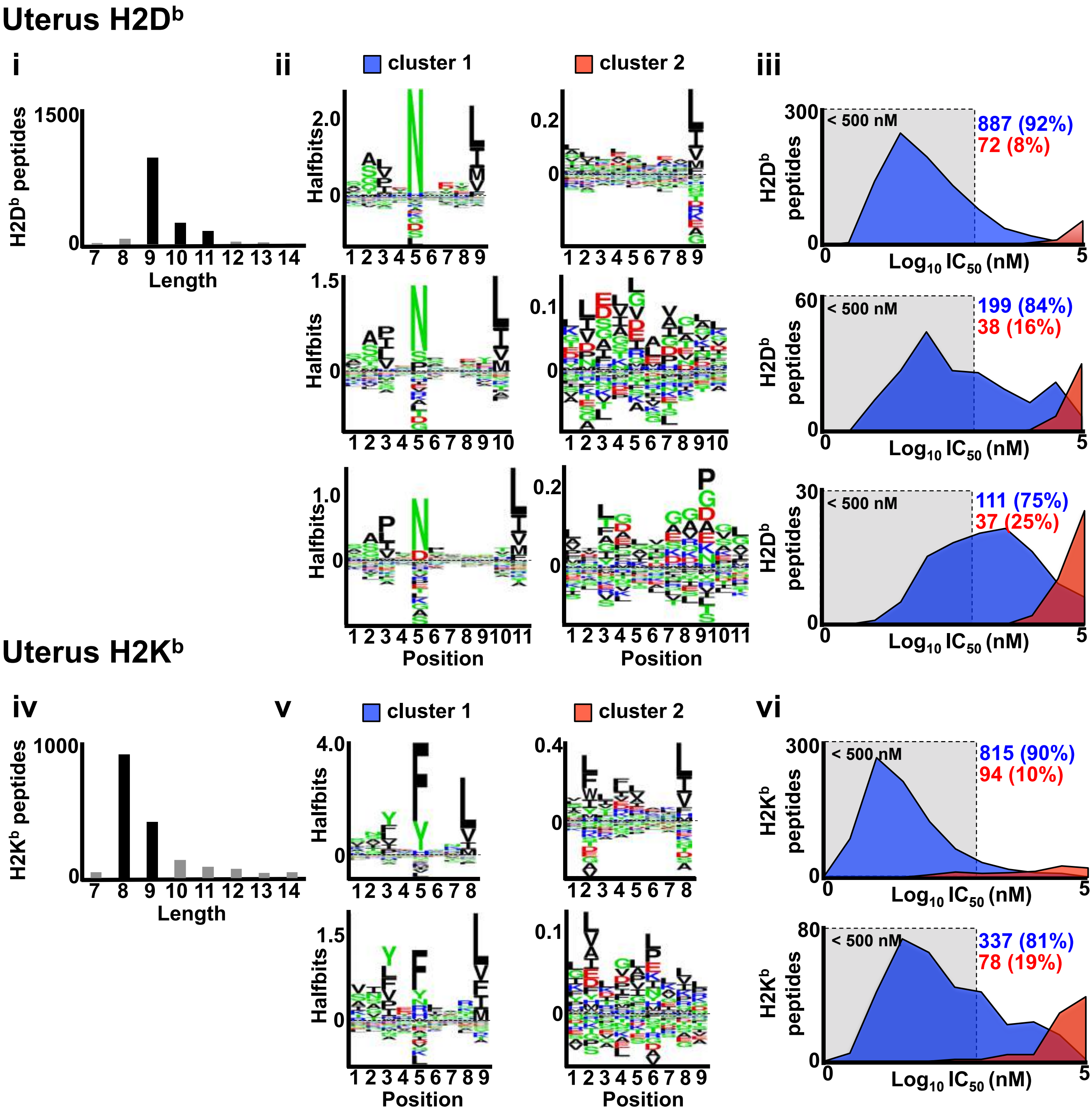

Supplementary Figure 2g. Analysis of all 7-14 mers identified at 1% FDR from uterus tissue. See legend of Supplementary Figure 2a for detailed description of the panels' figure.

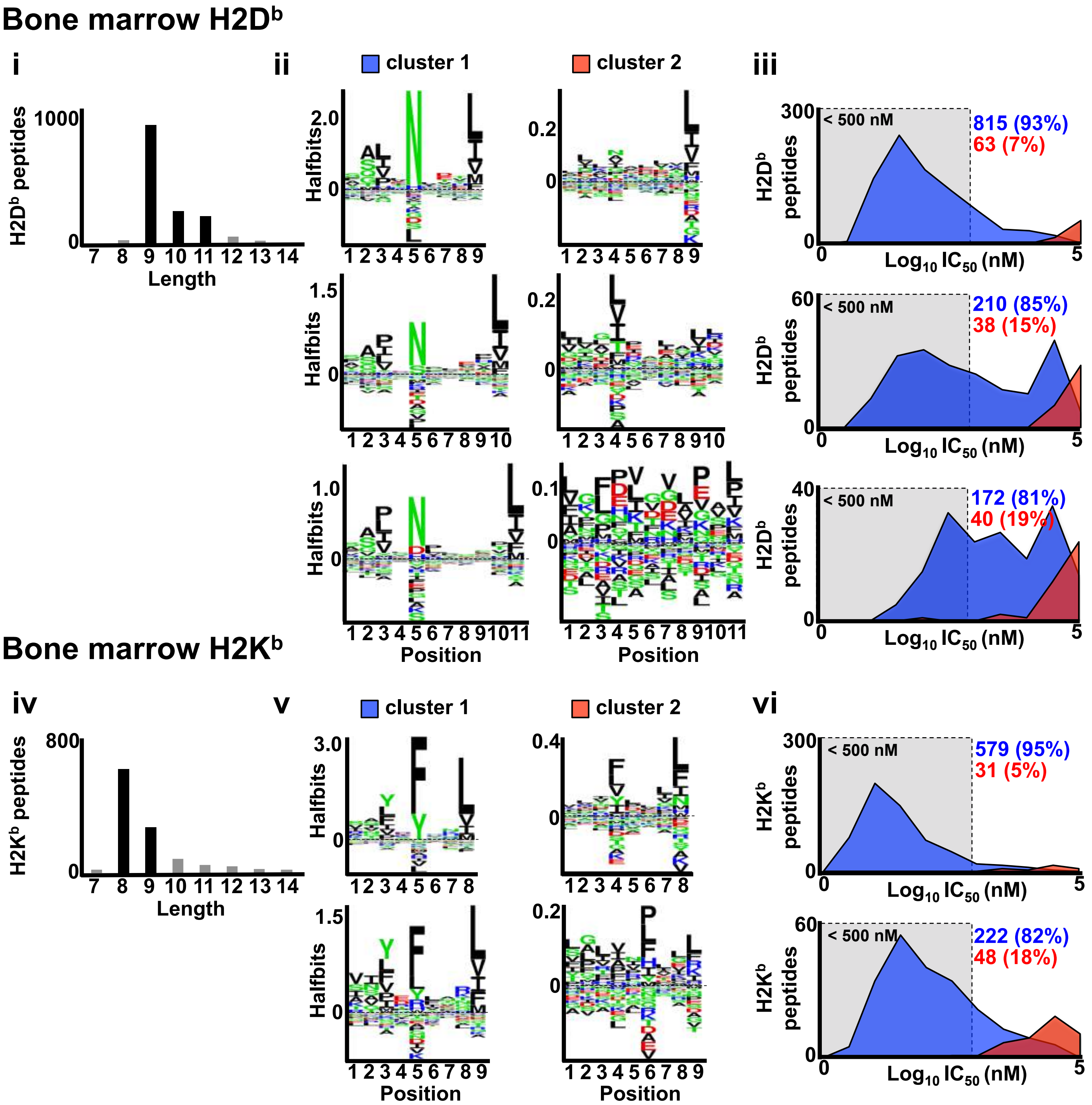

Supplementary Figure 2h. Analysis of all 7-14 mers identified at 1% FDR from bone marrow tissue. See legend of Supplementary Figure 2a for detailed description of the panels' figure.

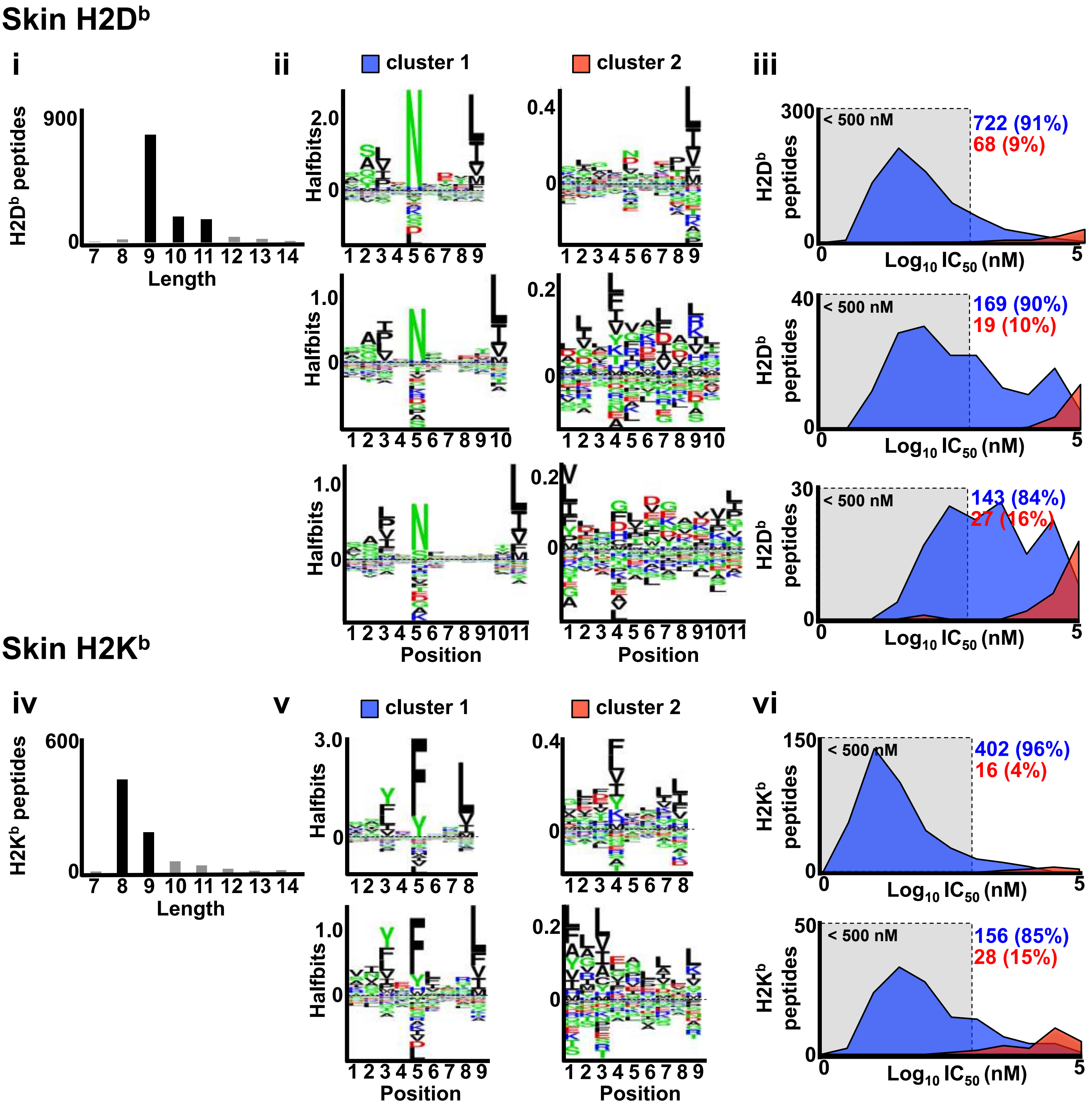

Supplementary Figure 2i. Analysis of all 7-14 mers identified at 1% FDR from skin tissue. See legend of Supplementary Figure 2a for detailed description of the panels' figure.

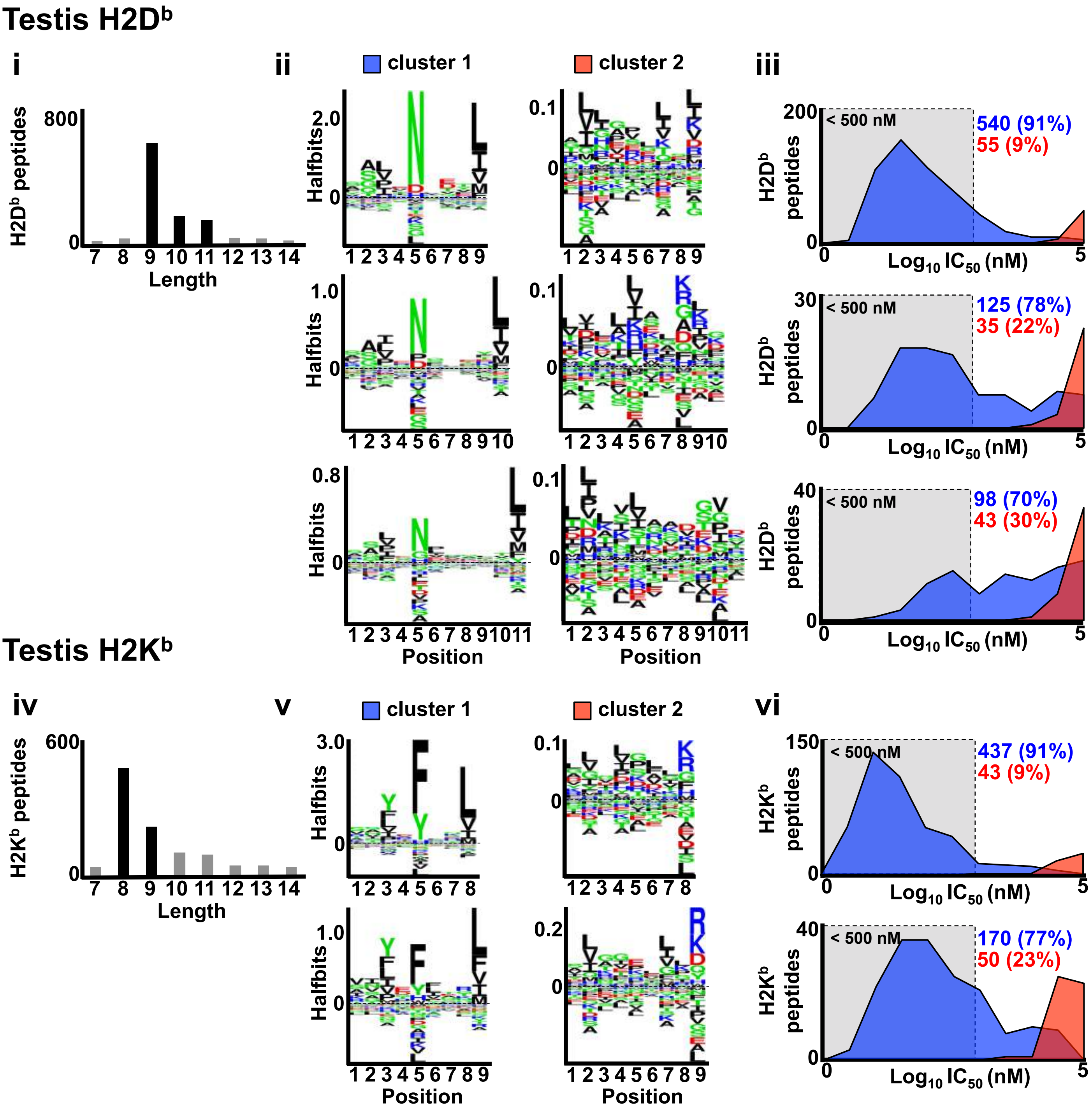

Supplementary Figure 2j. Analysis of all 7-14 mers identified at 1% FDR from testis tissue. See legend of Supplementary Figure 2a for detailed description of the panels' figure.

Adrenal gland H2D^b

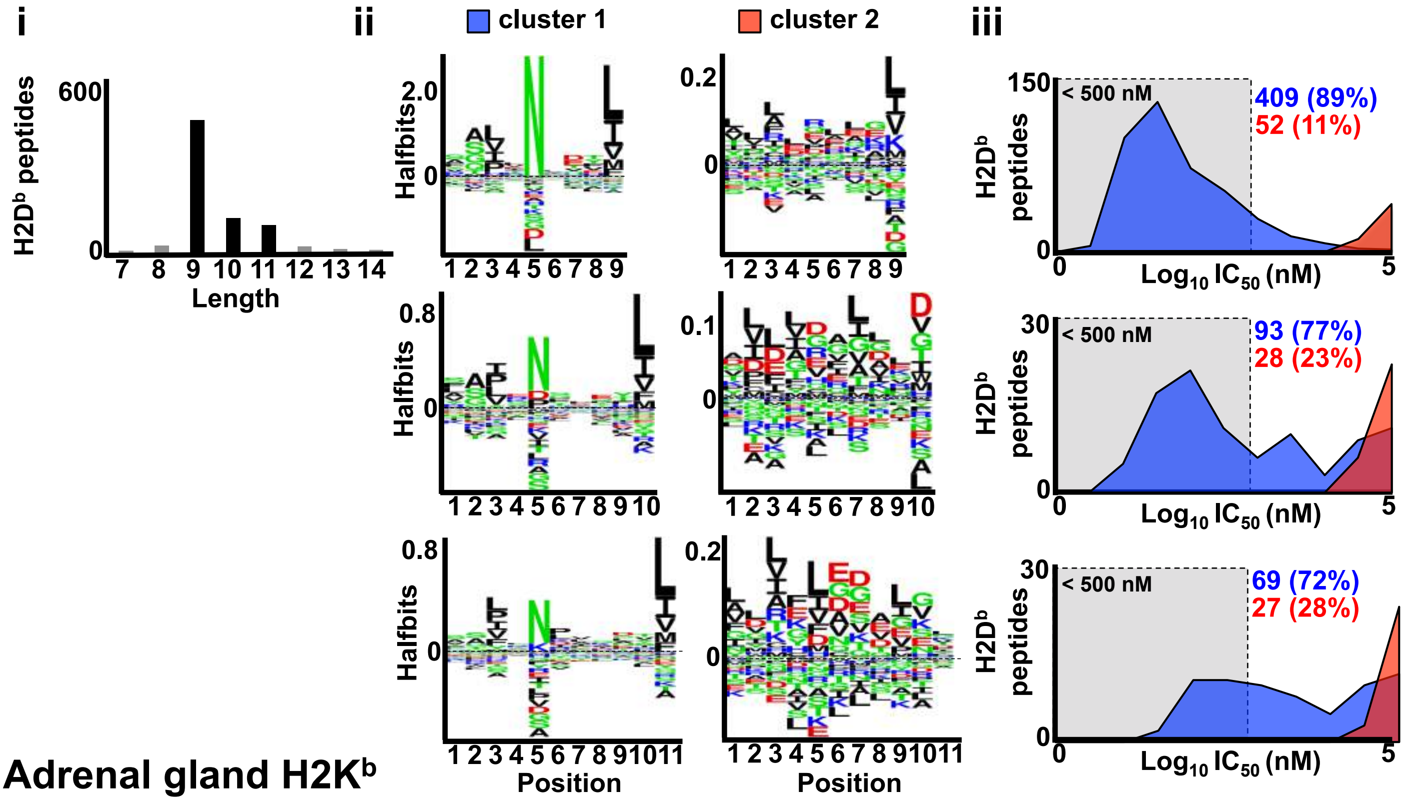

Adrenal gland H2K^b

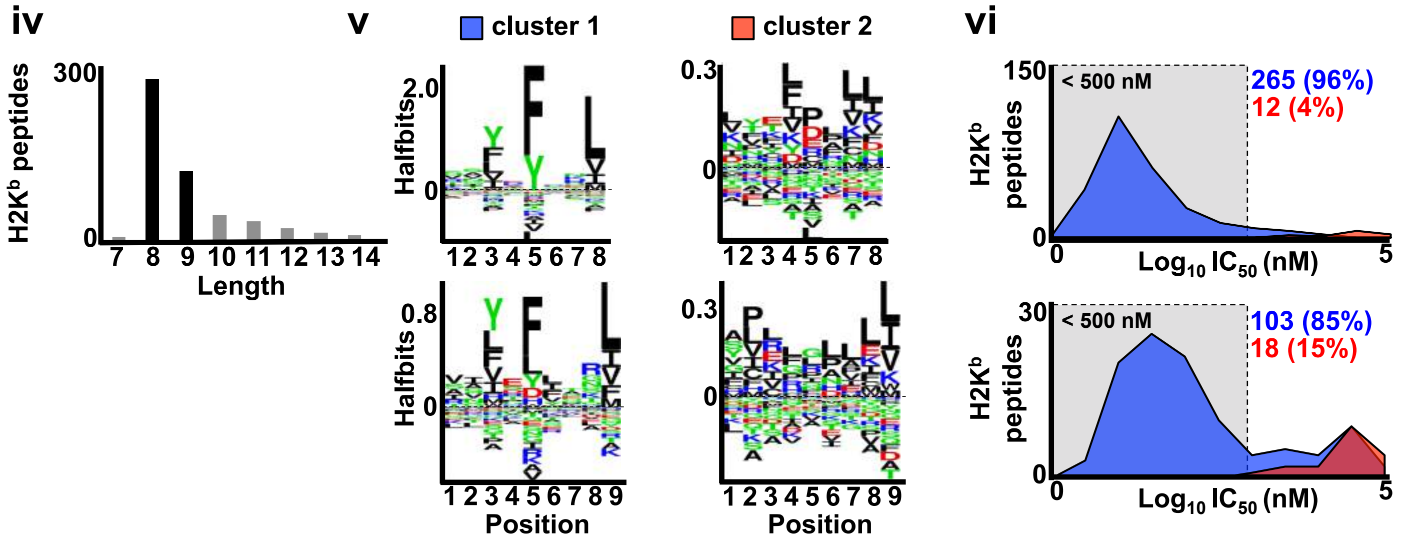

Supplementary Figure 2k. Analysis of all 7-14 mers identified at 1% FDR from adrenal gland tissue. See legend of Supplementary Figure 2a for detailed description of the panels' figure.

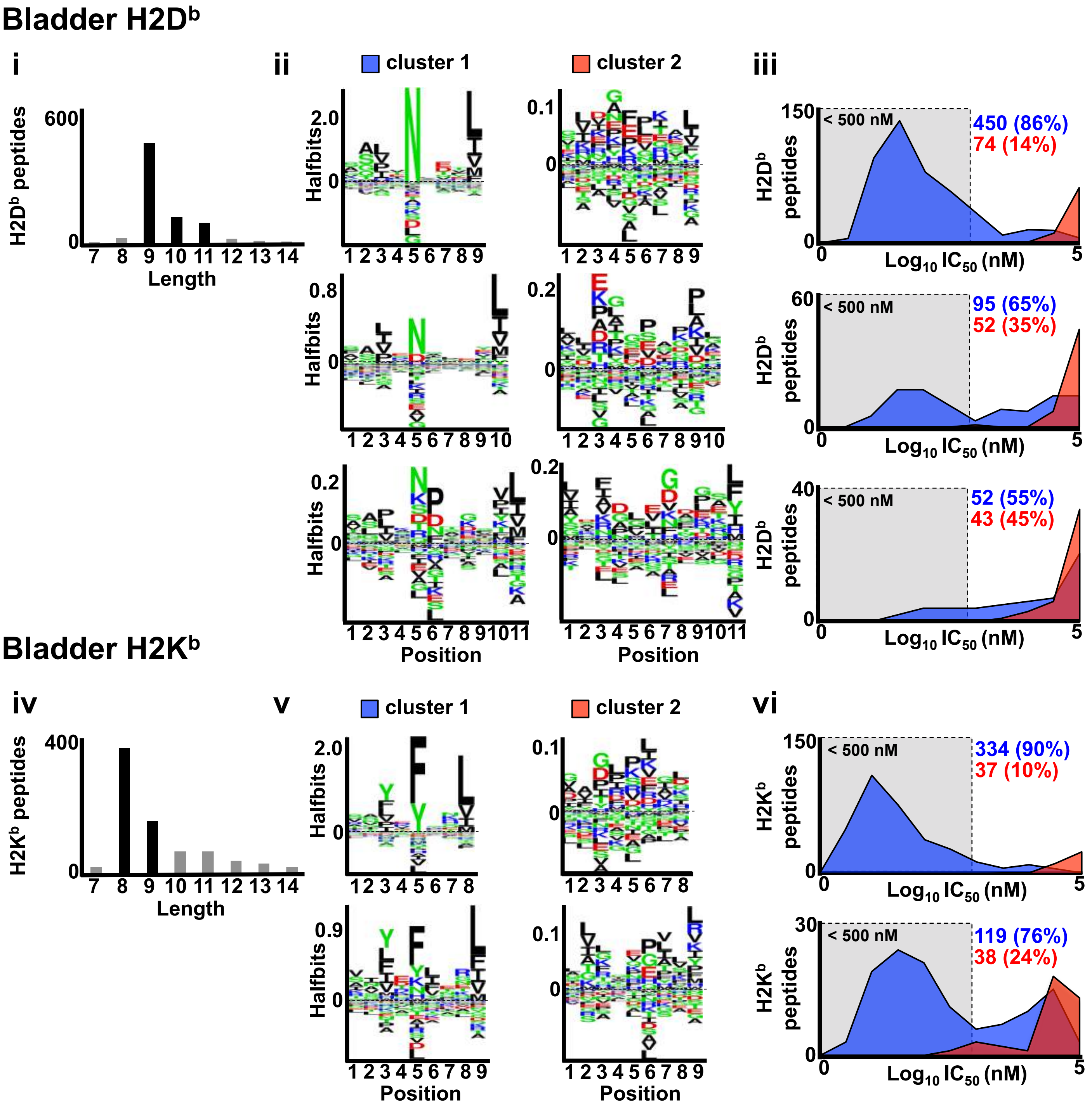

Supplementary Figure 2l. Analysis of all 7-14 mers identified at 1% FDR from bladder tissue. See legend of Supplementary Figure 2a for detailed description of the panels' figure.

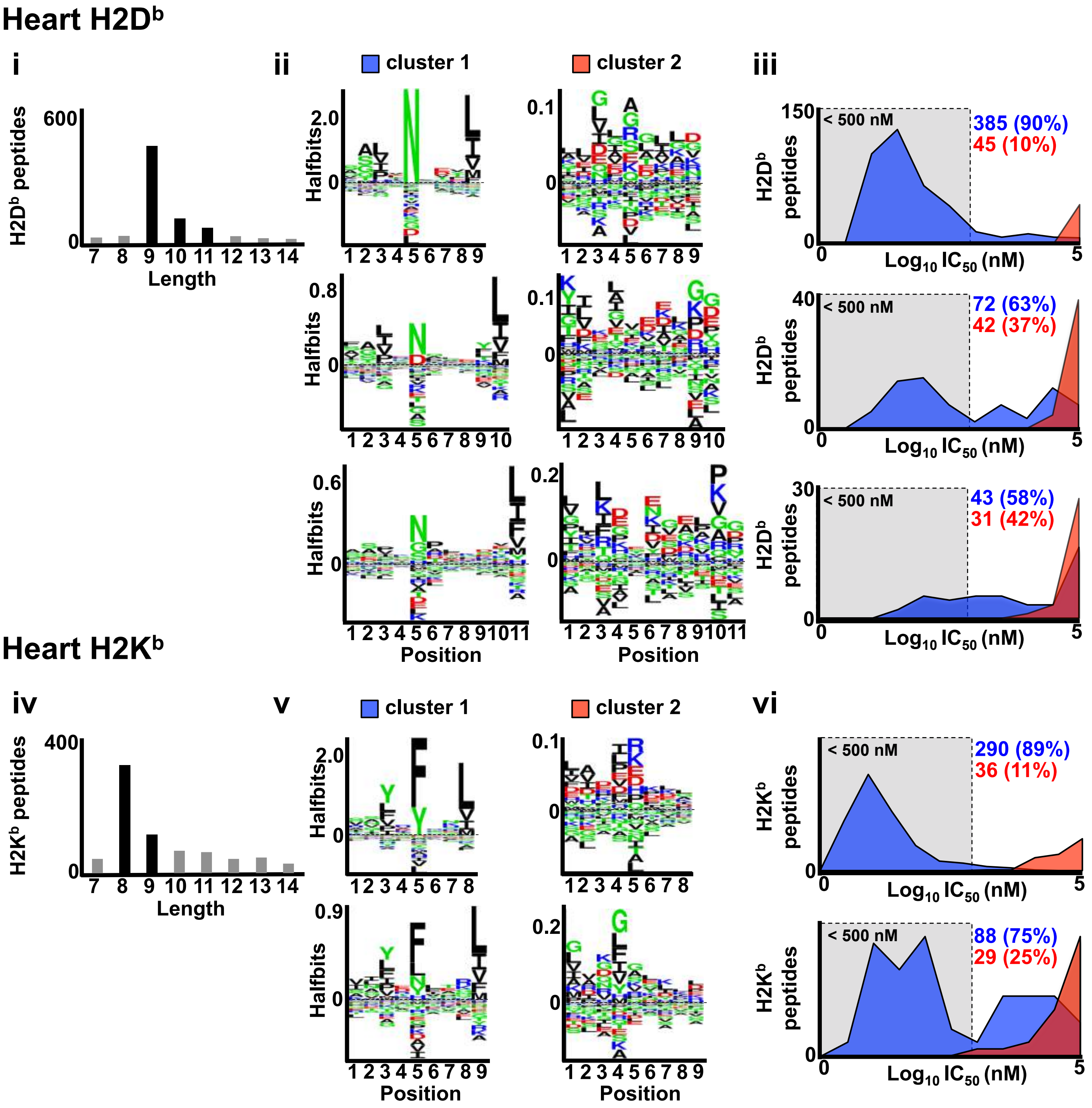

Supplementary Figure 2m. Analysis of all 7-14 mers identified at 1% FDR from heart tissue. See legend of Supplementary Figure 2a for detailed description of the panels' figure.

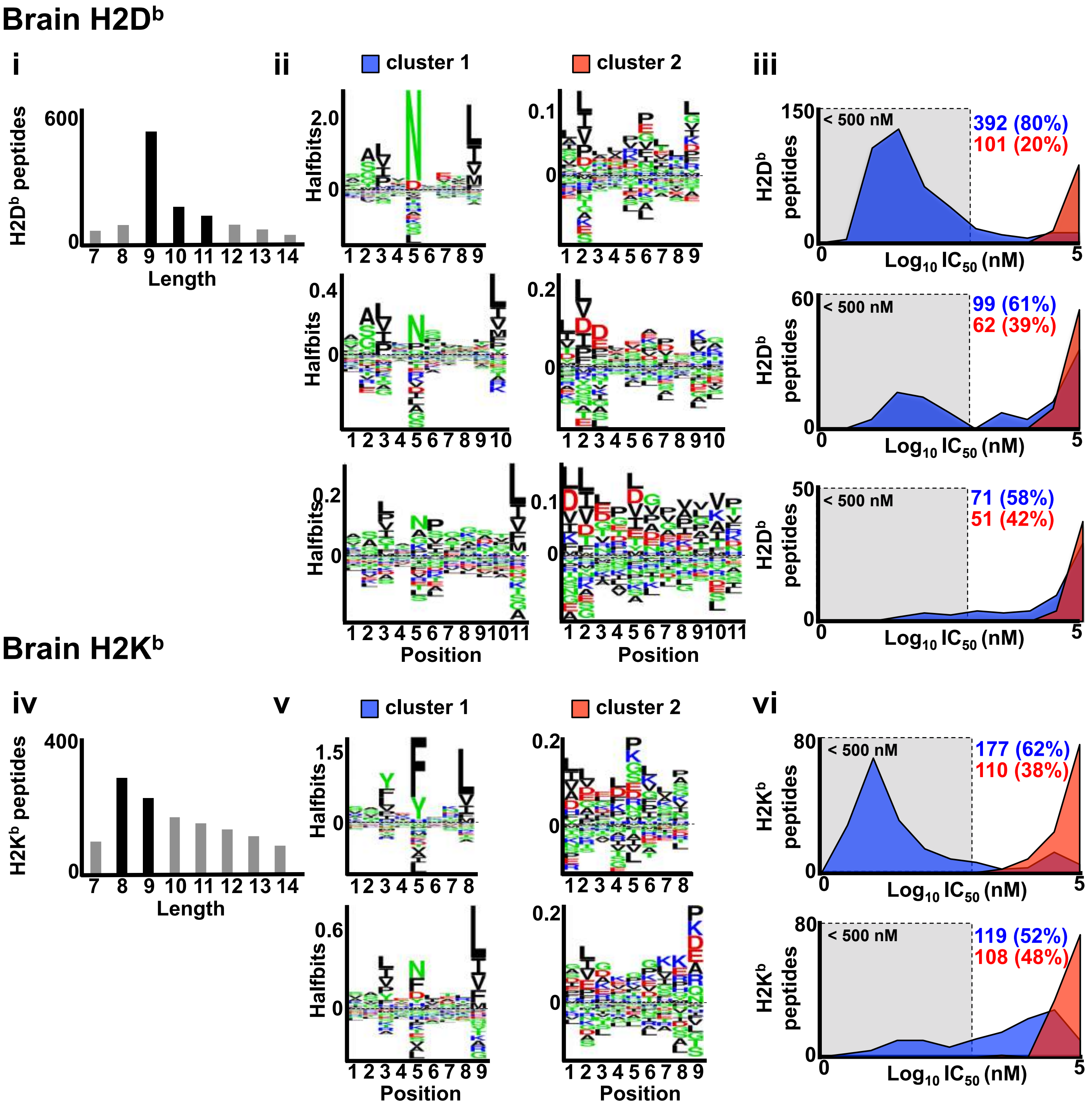

Supplementary Figure 2n. Analysis of all 7-14 mers identified at 1% FDR from brain tissue. See legend of Supplementary Figure 2a for detailed description of the panels' figure.

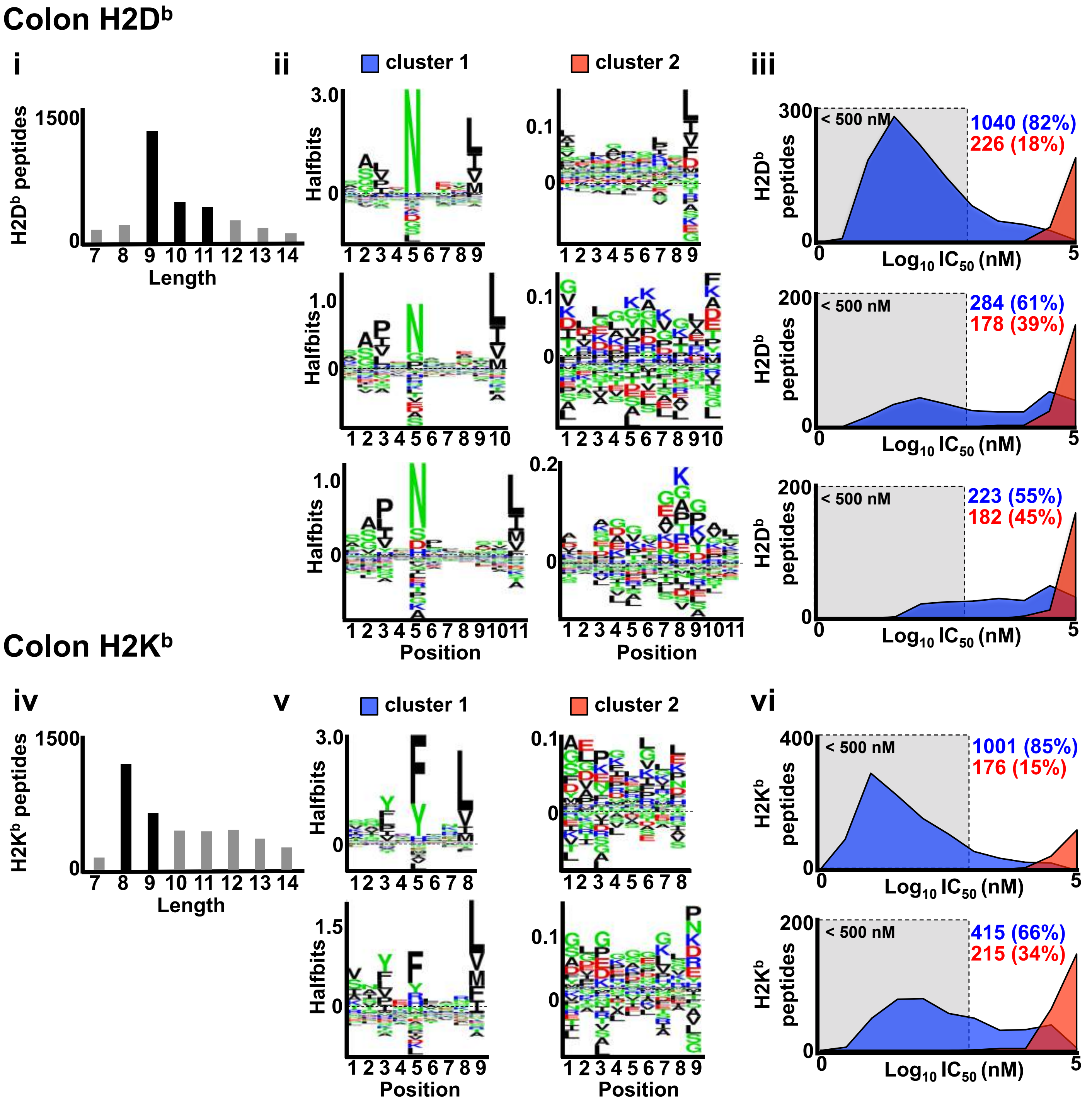

Supplementary Figure 2o. Analysis of all 7-14 mers identified at 1% FDR from colon tissue. See legend of Supplementary Figure 2a for detailed description of the panels' figure.

Ovary H2D^b

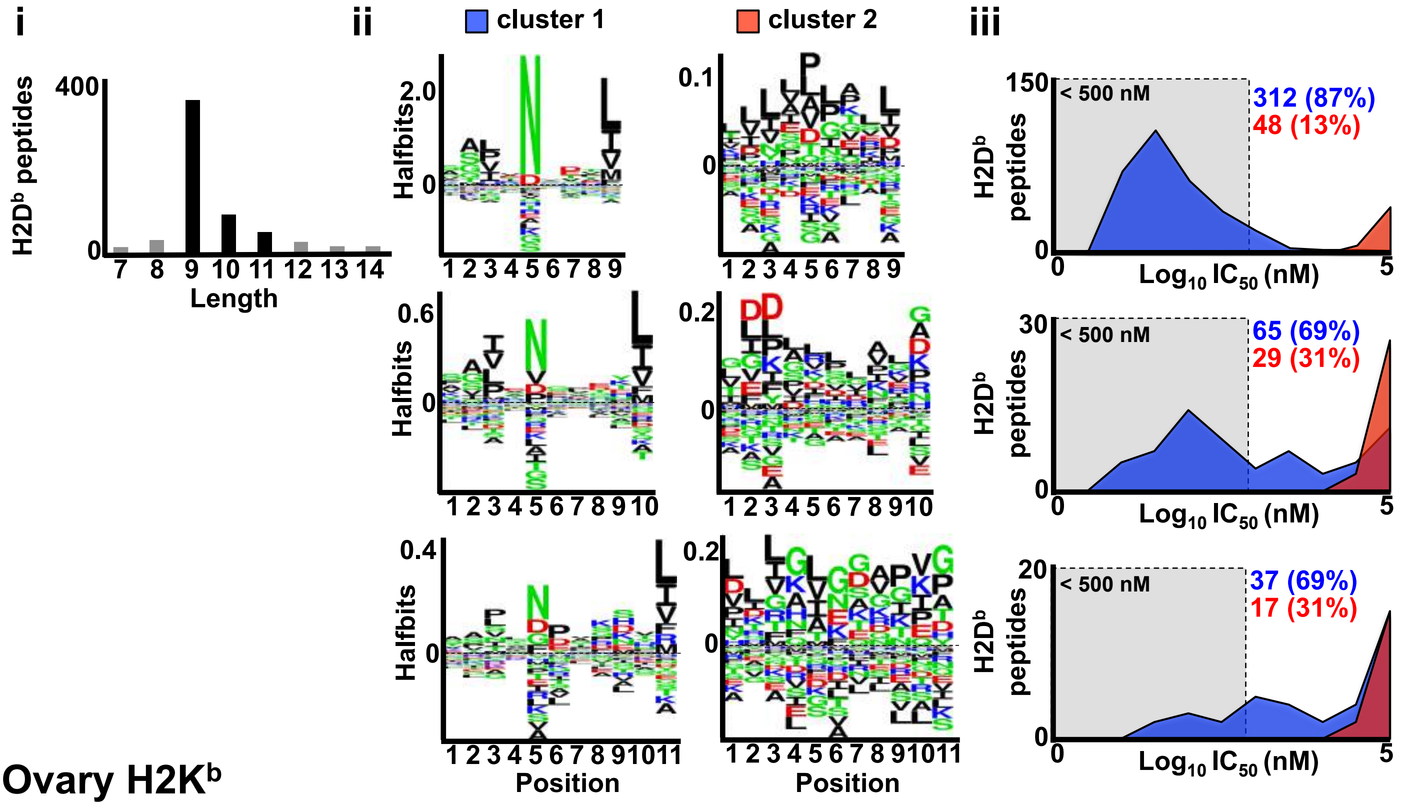

Ovary H2K^b

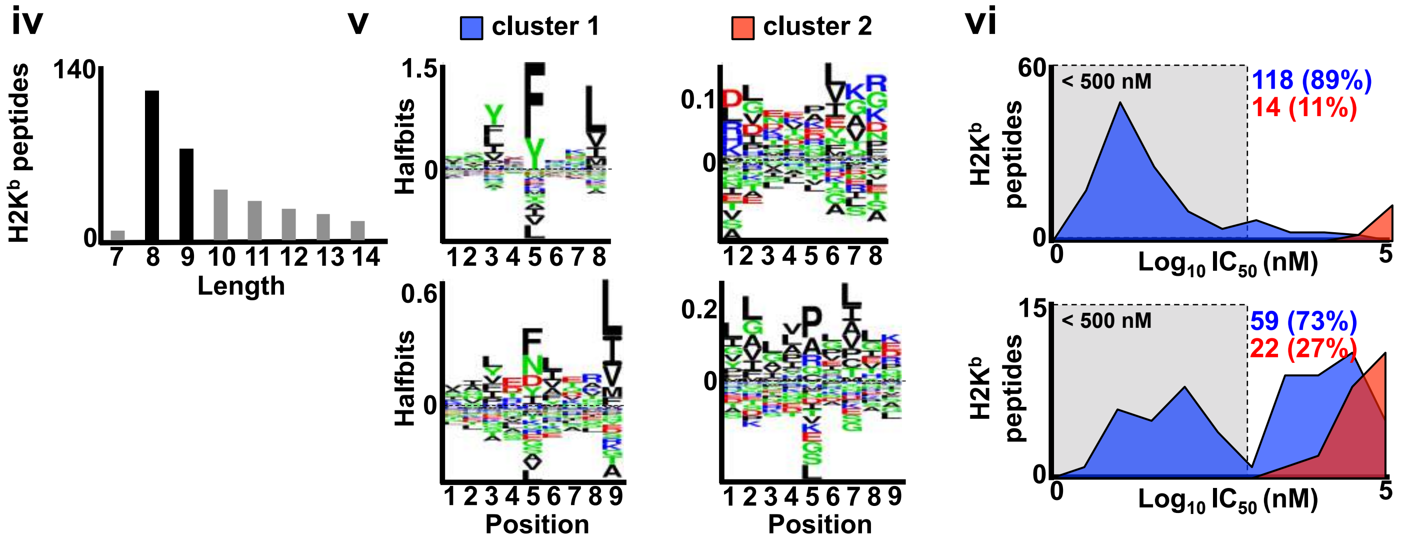

Supplementary Figure 2p. Analysis of all 7-14 mers identified at 1% FDR from ovary tissue. See legend of Supplementary Figure 2a for detailed description of the panels' figure.

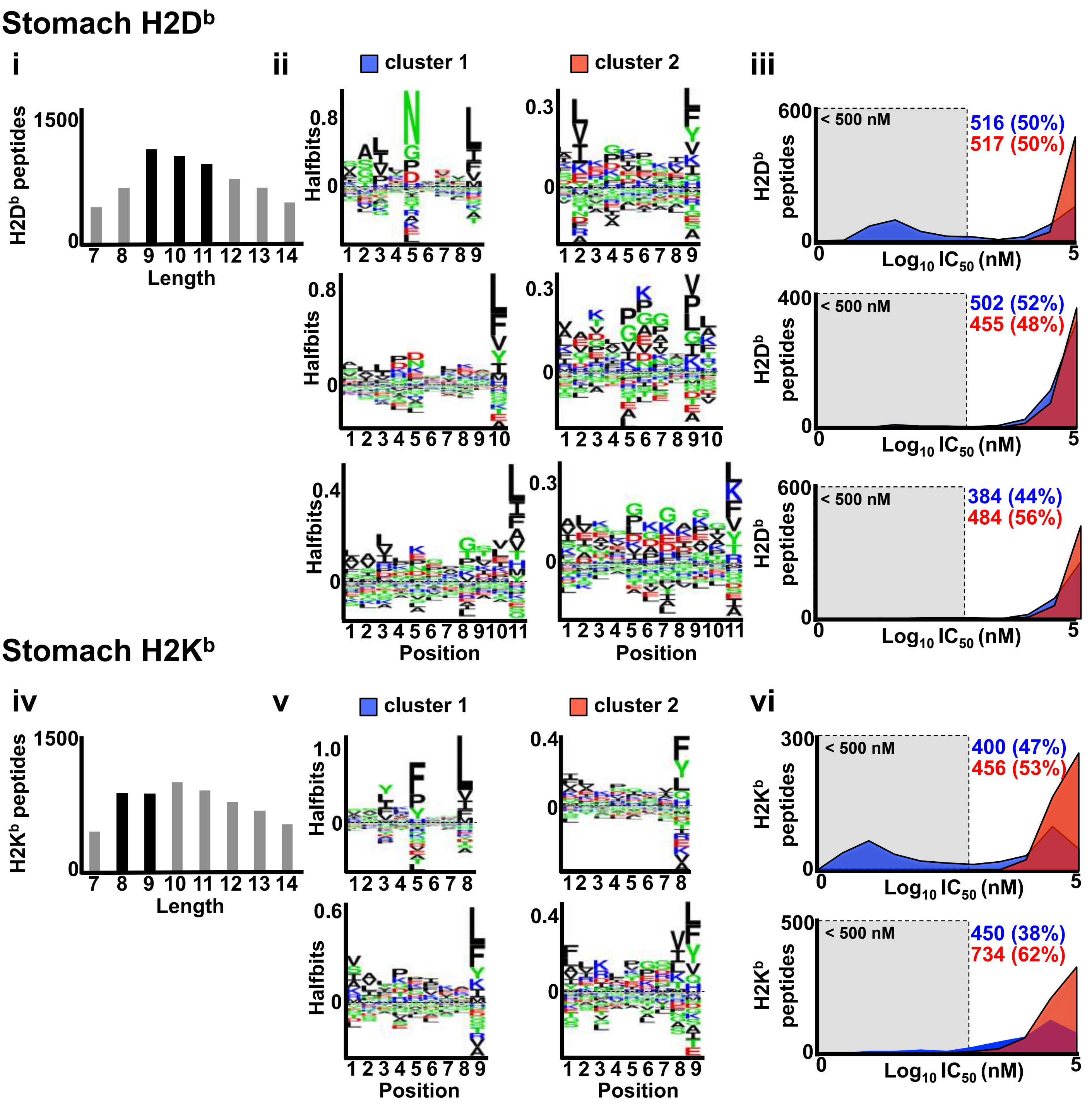

Supplementary Figure 2q. Analysis of all 7-14 mers identified at 1% FDR from stomach tissue. See legend of Supplementary Figure 2a for detailed description of the panels' figure.

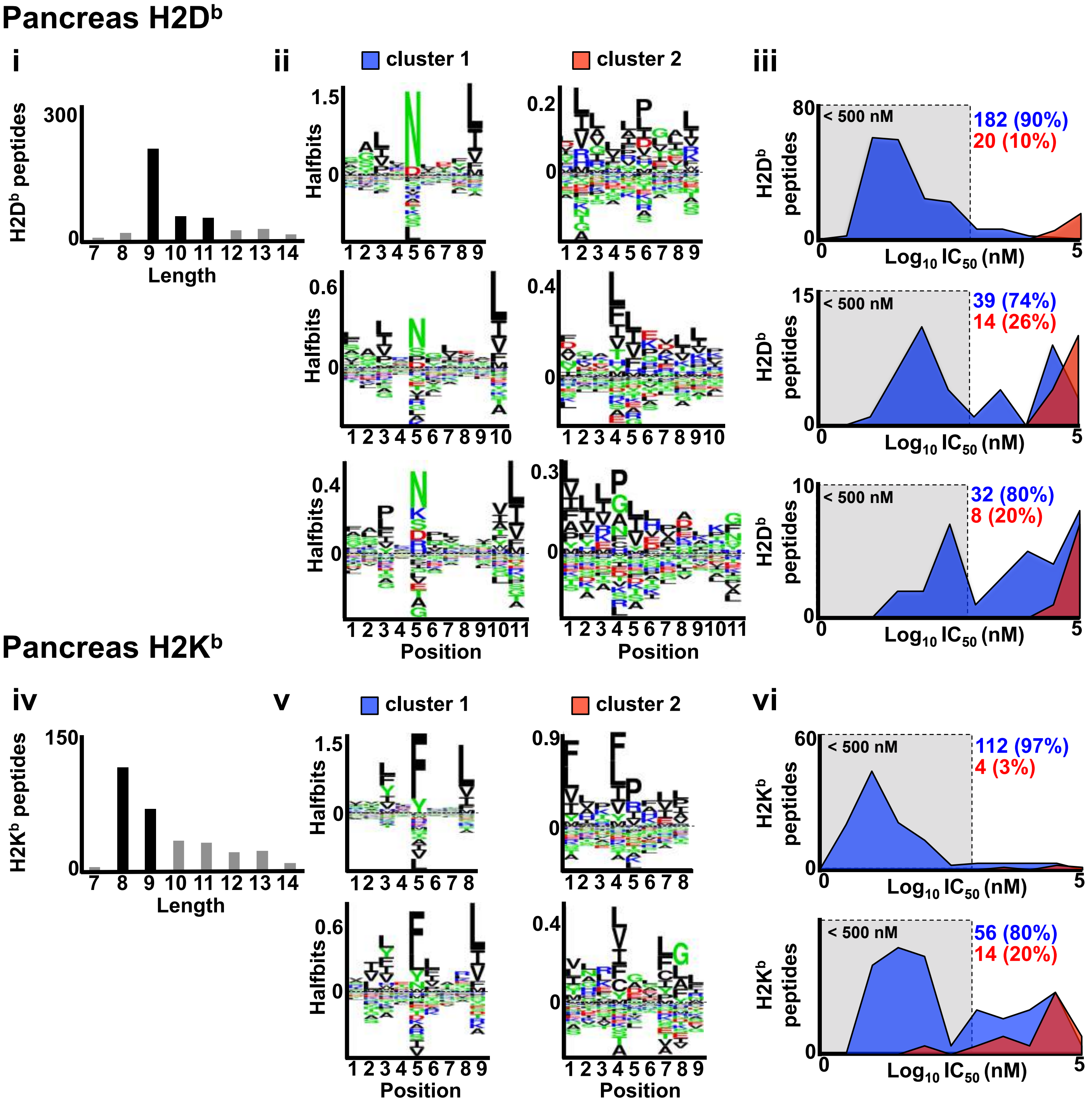

Supplementary Figure 2r. Analysis of all 7-14 mers identified at 1% FDR from pancreas tissue. See legend of Supplementary Figure 2a for detailed description of the panels' figure.

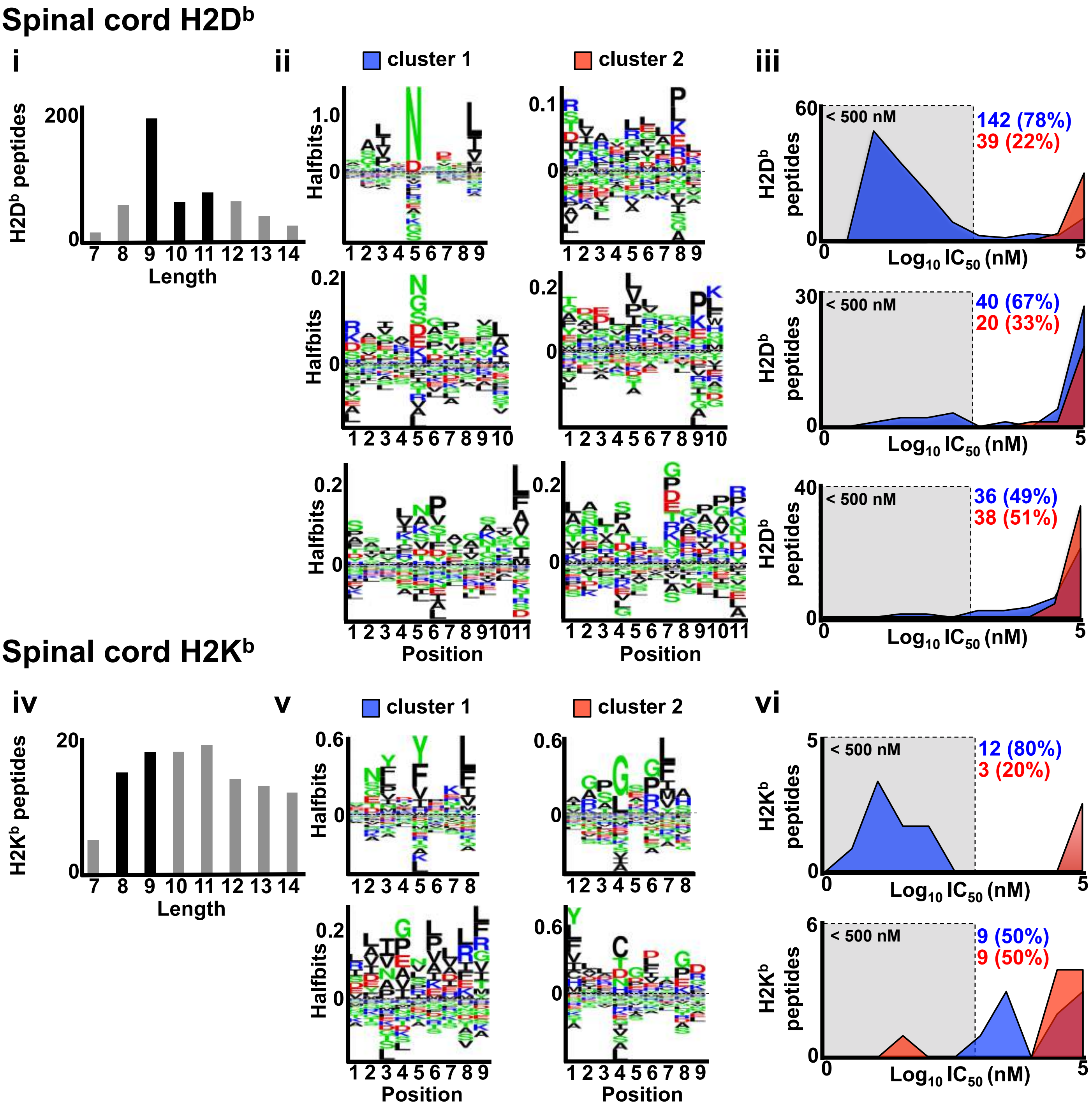

Supplementary Figure 2s. Analysis of all 7-14 mers identified at 1% FDR from spinal cord tissue. See legend of Supplementary Figure 2a for detailed description of the panels' figure.

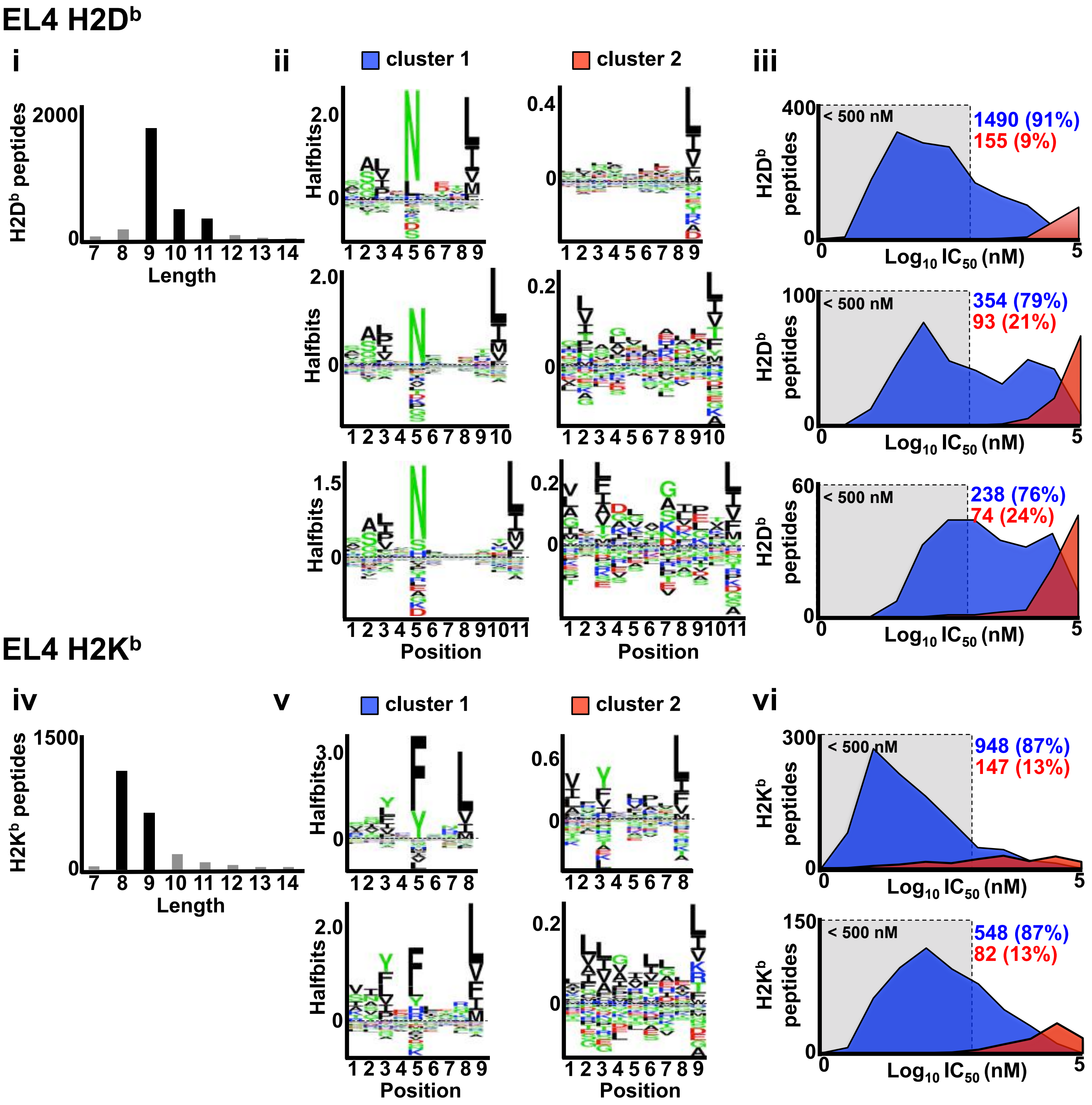

Supplementary Figure 2t. Analysis of all 7-14 mers identified at 1% FDR from EL4 cells. See legend of Supplementary Figure 2a for detailed description of the panels' figure.

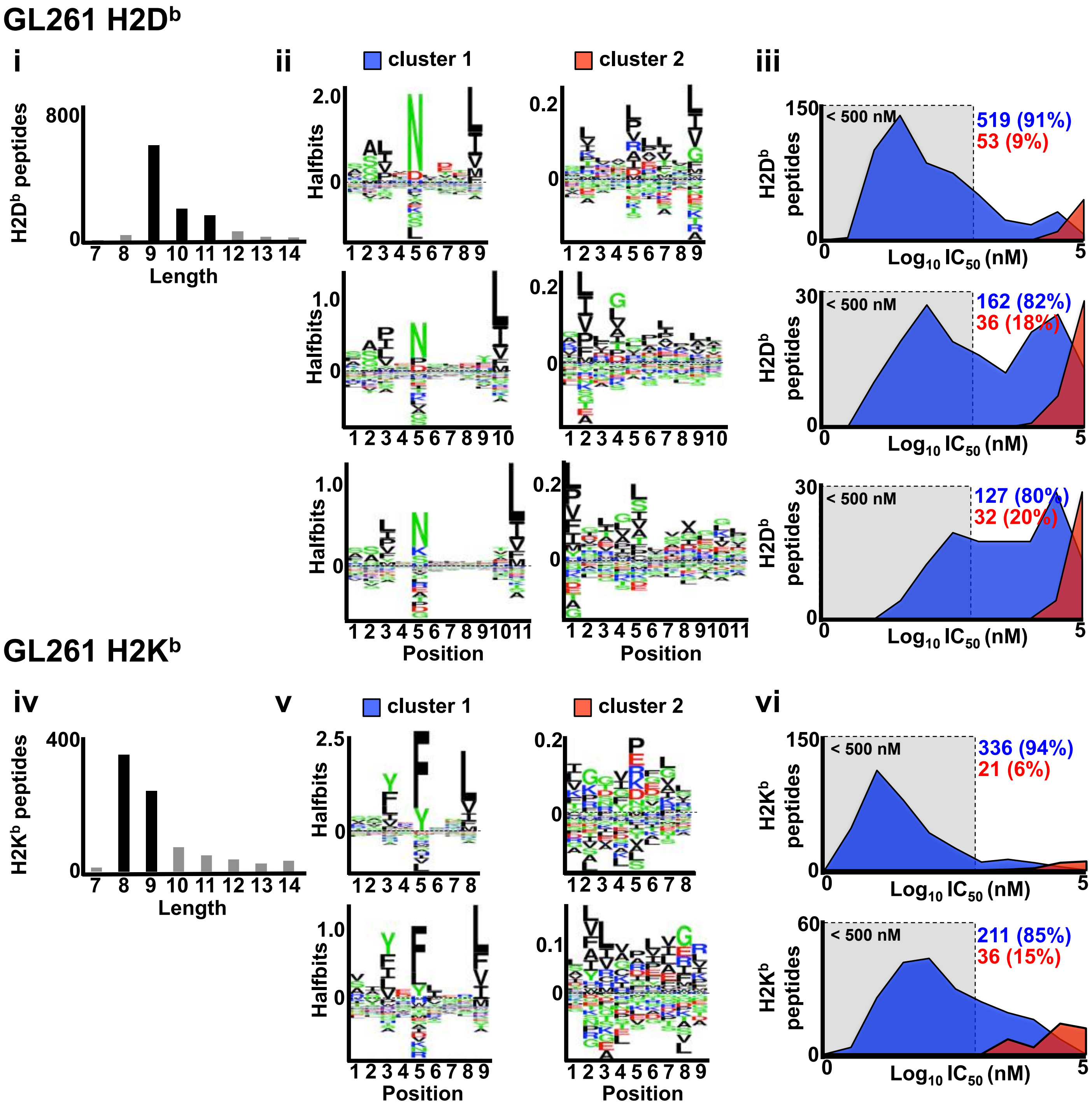

Supplementary Figure 2u. Analysis of all 7-14 mers identified at 1% FDR from GL261 cells. See legend of Supplementary Figure 2a for detailed description of the panels' figure.

LLC1 H2D^b

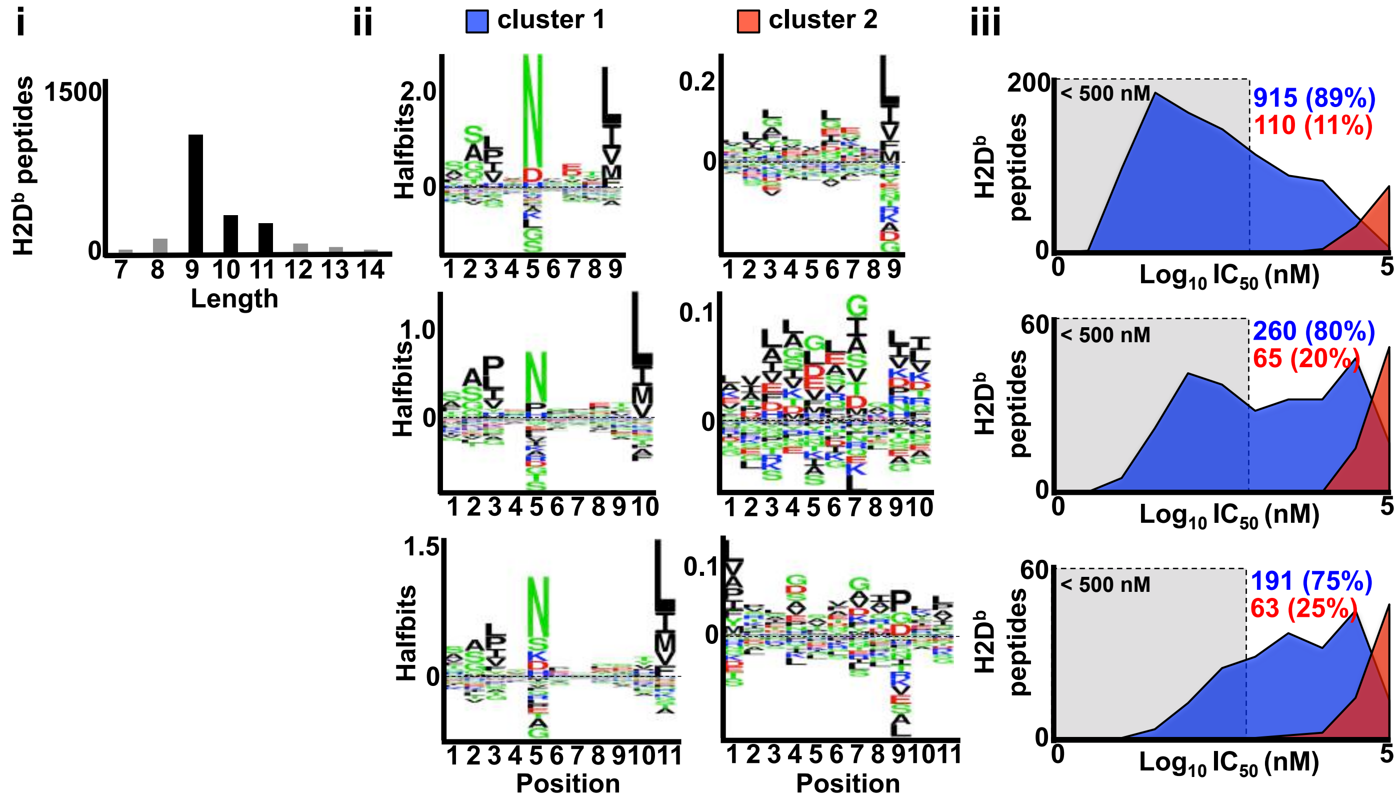

Supplementary Figure 2v. Analysis of all 7-14 mers identified at 1% FDR from LLC1 cells. See legend of Supplementary Figure 2a for detailed description of the panels' figure. Note that H2K^b peptides were removed from the analysis.

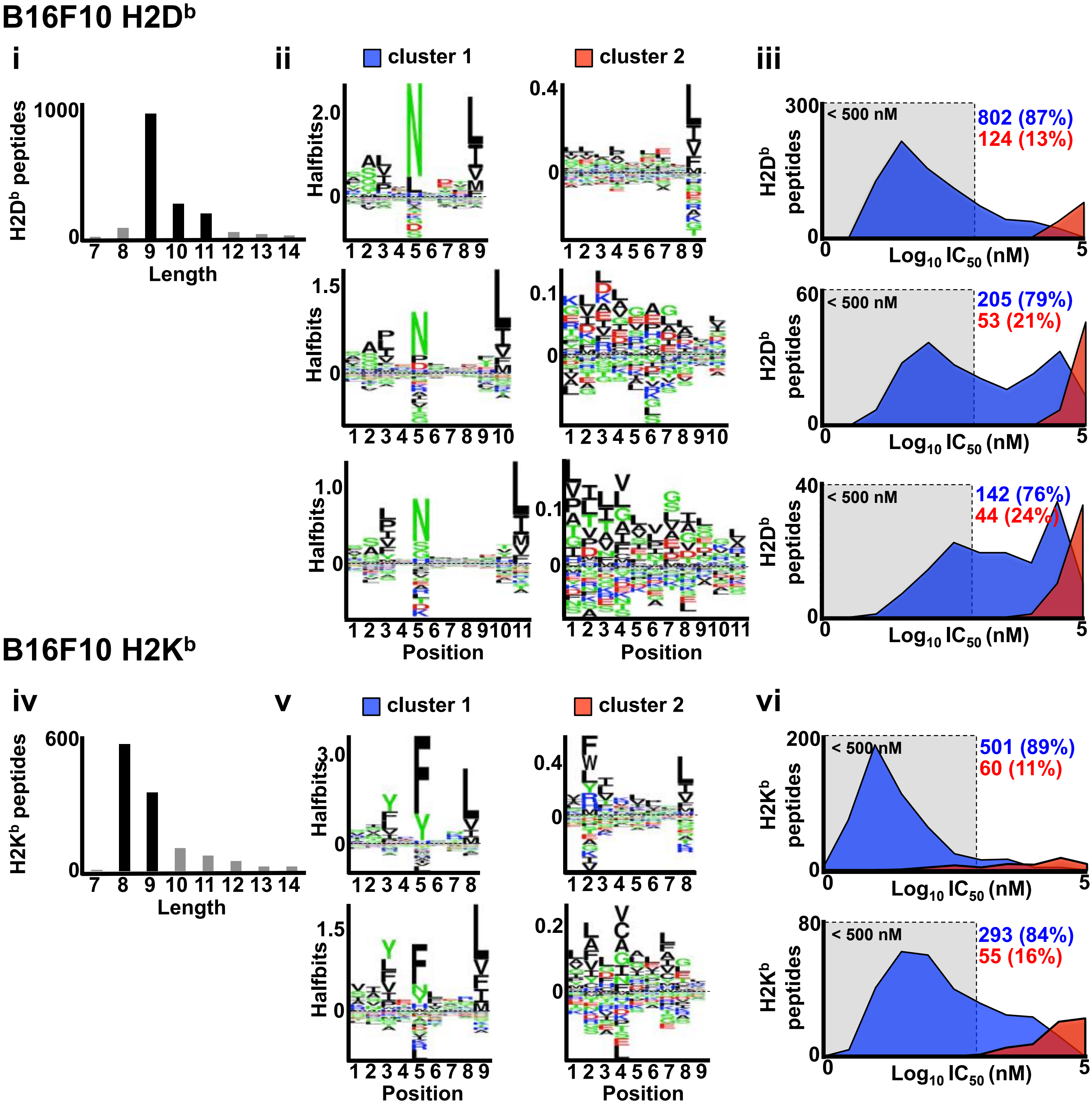

Supplementary Figure 2w. Analysis of all 7-14 mers identified at 1% FDR from B16F10 cells. See legend of Supplementary Figure 2a for detailed description of the panels' figure.

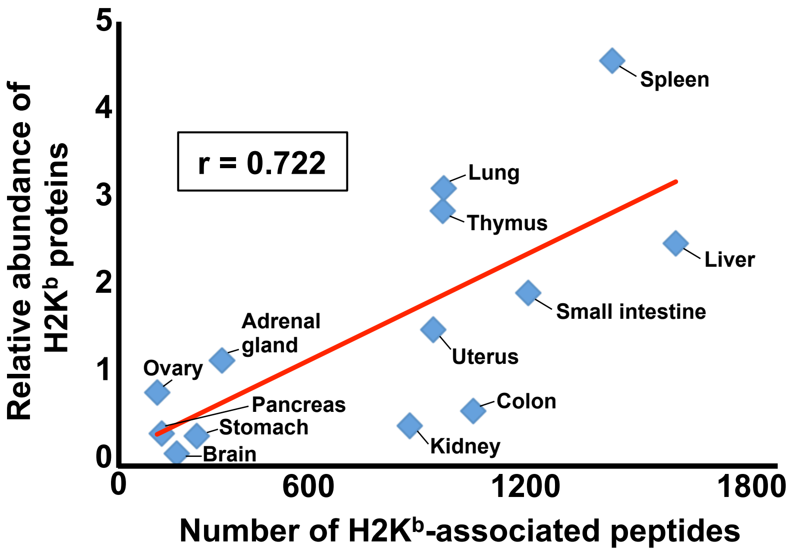

Supplementary Figure 3. Pearson correlation between the number of high-confidence H2K^b-associated peptides identified per tissue (x-axis) and the abundance of H2K^b proteins quantified per tissue (y-axis). Abundance of H2K^b proteins were obtained from a previous study (Geiger et al., 2013)[43]. Numbers of H2K^b-associated peptides are in Figure 2c. Note that H2D^b proteins were not consistently quantified across tissues in Geiger et al. (2013) and were therefore not included in this graph.

a

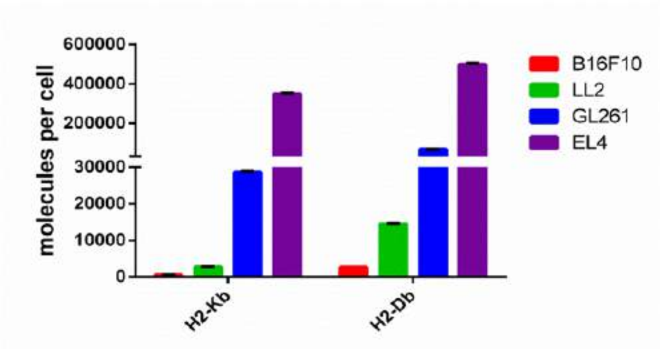

	H2-Kb	H2-Db
**B16F10**	500	2700
**LLC1**	2700	14500
**GL261**	28700	67000
**EL4**	347300	499400

b

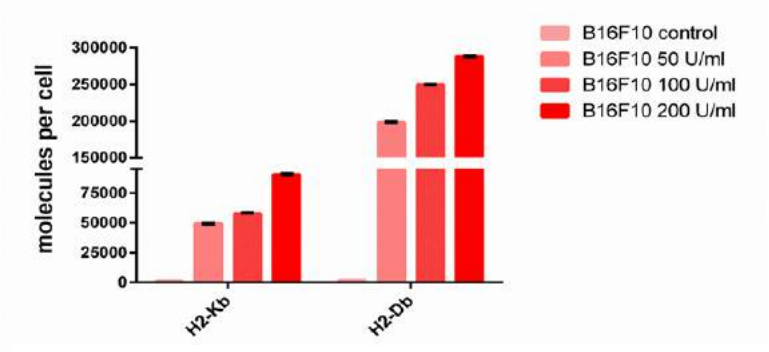

	H2-Kb	H2-Db
**control**	1000	1650
**50 U/ml**	49000	198000
**100 U/ml**	57600	249600
**200 U/ml**	90300	288400

c

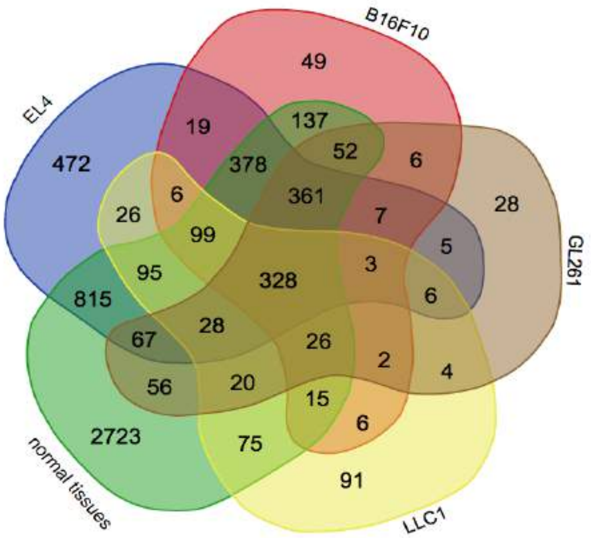

Supplementary Figure 4. Comparison of high-confidence H2D^b/K^b peptides identified from normal tissues and tumor cell lines. (**a**) Cell surface H2D^b and H2K^b proteins were quantified for the indicated cell lines using the flow cytometry-based QIFIKIT assay. (**b**) B16F10 cells were treated at various concentration of IFNγ (U/mL) and the abundance of H2K^b and H2D^b proteins was quantified using the flow cytometry-based QIFIKIT assay. Measured number of cell surface molecules/proteins per cell is indicated in the boxes below the histograms. (**c**) Venn diagram showing the overlap of peptides between those identified in all the healthy tissues and those identified from the four tumor cell lines. The venn diagram can be reproduced using the data in Supplementary Table 4 and Supplementary Table 5, and the following link: http://bioinformatics.psb.ugent.be/webtools/Venn/.
